# Enhancing Strategic Agility and Real Time Decision-Making in the Technology Sector: Exploring the Role of AI and e-HRM Systems

**DOI:** 10.12688/f1000research.167708.1

**Published:** 2025-11-10

**Authors:** Tom Ongesa Nyamboga

**Affiliations:** 1 Business Administration, Kampala International University - Western Campus, Bushenyi, Western Region, Uganda

**Keywords:** Technology Industry, Artificial Intelligence, e-HRM, Strategic Agility, Real Time Decision Making

## Abstract

We are living in an era of rapid transformation in the technology sector, where success depends on shifting from traditional static strategies to dynamic models that emphasize strategic agility and real-time decision making as shown in Figure 1. Despite ongoing digital transformation, a critical gap remains in understanding how predictive Artificial Intelligence (AI) and electronic Human Resource Management (e-HRM) tools integrate into policy frameworks that future-proof organizations against disruption. This systematic literature review provides an evidence-based analysis of how digital AI and e-HRM platforms can be utilized to enhance strategic agility and real-time decision making in the technology industry. Through qualitative and thematic synthesis analysis of **s**tudies published between 2015 and 2025, the review finds that AI and e-HRM significantly strengthen strategic agility. However, challenges such as data privacy concerns, algorithmic bias, integration difficulties, and resistance to AI adoption may limit scalability and ethical use. While AI-driven tools offer strong potential for improving agility, addressing ethical, technical, and organizational challenges is crucial to fully realize their benefits. The findings highlight the need for policies supporting ethical AI practices, robust data governance, and workforce readiness to ensure effective integration of AI tools and strengthen organizational agility and real time decision-making.

## Introduction

In the technology sector, strategic agility and effective decision-making are essential for navigating rapid innovation, market volatility, and post-pandemic disruptions ( Nosike, et al., 2023). The integration of AI and e-HRM has emerged as a key enabler of these capabilities ( Rana & Kaur, 2024). AI supports real-time data analysis, predictive modeling, and intelligent automation, allowing organizations to respond quickly and accurately to emerging challenges ( Paramesha, et al., 2024). At the same time, e-HRM digitizes core HR functions such as talent acquisition, performance management, and workforce planning, ensuring that HR strategies stay aligned with evolving organizational goals ( Al Samman & Al Obaidly, 2024, January; Sani, et al., 2024). Together, AI and e-HRM not only enhance operational efficiency but also build adaptive capacity, enabling better decision-making under uncertainty ( Sheikh, et al., 2025; Al-Surmi, et al., 2022). This combined approach empowers leaders to forecast talent needs, foster continuous learning, and match workforce skills with shifting strategic priorities ( Maheswari, et al., 2024; Serafini & Szamosi, 2025; Rane, et al., 2024a; Bannikov, et al., 2024).

Strategic agility in the technology industry refers to an organization’s ability to quickly adapt to technological changes, market fluctuations, and competitive pressures ( Poi & Lebura, 2022), while real-time decision-making involves instantly processing and acting on data to maintain strategic focus and operational efficiency ( Olayinka, 2021). These capabilities are critical in high-velocity environments where delayed responses can result in lost market share or slowed innovation ( Ridwan, 2025; de Diego Ruiz, 2023; George, 2023). Many firms, however, struggle with outdated or disconnected HR systems that lack the capacity to provide timely insights into workforce trends, talent availability, and performance metrics ( Minbaeva, 2021; Kakepota & Atif, 2024). This limits their ability to respond strategically and make rapid, informed decisions. AI and e-HRM help close this gap by enabling intelligent automation, predictive analytics, and digital talent management, supporting proactive workforce planning, continuous performance tracking, and flexible learning. Together, these tools improve both agility and the accuracy of decision-making ( Alqarni, et al., 2023; Roul, et al., 2024).

In advanced economies, strategic agility and real-time decision-making are key to maintaining competitiveness in the technology sector ( Mustafa, et al., 2025; Neiroukh, et al., 2024, January). Rapid digital transformation in countries like the United States, Germany, South Korea, and Japan has driven tech firms to constantly evolve, innovate, and react quickly to market changes ( Yun, et al., 2025; Park & Shintaku, 2022; Schaede & Shimizu, 2022). In the U.S., companies such as Google and Amazon have embedded strategic agility through decentralized structures supported by real-time data analytics, allowing them to respond rapidly to consumer trends and disruptions ( Gatlin, 2024; Emma, 2024a). Amazon, for instance, used AI-powered logistics and workforce systems during the COVID-19 pandemic to adapt operations in real time and meet changing demand efficiently ( Shobhana, 2024; Munuhwa & Chibaro, 2024). In Germany, SAP has integrated AI into its e-HRM platforms to predict workforce needs and run scenario simulations, enhancing agility and strategic planning ( Lemola, 2024). Samsung in South Korea uses AI tools to track performance and reassign talent across innovation teams as needed, a crucial capability in the fast-paced electronics sector ( Schäch, 2025). These cases show how AI and e-HRM are helping firms in advanced economies strengthen both responsiveness and resilience.

Despite notable advancements, key challenges remain in fully leveraging AI and e-HRM for strategic agility. Many firms in advanced economies still contend with fragmented HR systems, skill mismatches, and inconsistent application of predictive workforce analytics ( Pandey, et al., 2023). Integrating AI into HR functions without compromising employee autonomy and engagement continues to be a major hurdle ( Vishwanath & Vaddepalli, 2023; Honnamane & Girish, 2024). The absence of unified platforms that align HR data with strategic goals often results in inefficiencies and delayed responses during uncertainty ( Okon, et al., 2024). Although digital tools are widely available, gaps in customization and workforce readiness hinder their full adoption ( Trenerry, et al., 2021). This study examines how AI-integrated e-HRM systems can close these gaps by aligning technical capabilities with the social dynamics of human capital. It offers a framework to support more agile, real-time decision-making, providing insights for firms seeking resilience and competitiveness in an increasingly volatile and complex digital landscape.

In developing economies, the drive for strategic agility and real-time decision-making in the technology sector is growing, though the progress is uneven due to infrastructure gaps, limited institutional support, and human capital constraints ( Jaafar, et al., 2025; Aderibigbe, et al., 2023). Countries such as India, Kenya, Brazil, and Nigeria are advancing in their adoption of digital tools to boost business responsiveness. In India, firms like Infosys and Wipro use AI in their e-HRM systems to support workforce planning and upskilling, allowing for agile talent deployment ( Pandey, et al., 2023, 2024). Kenya’s tech ecosystem, particularly Nairobi’s Silicon Savannah, leverages mobile-based e-HRM platforms to enable data-driven labor and operational decisions in real time ( Mutegi, et al., 2023, May; Muathe, et al., 2022).

Despite these advancements, challenges remain. Many firms lack access to advanced e-HRM systems and face underinvestment in AI. Issues such as fragmented HR data, low digital literacy among HR staff, and weak predictive analytics limit effective decision-making. Cultural resistance to decentralized decision-making and misalignment between national innovation policies and enterprise-level HR reforms also hinder agility. This study addresses these issues by highlighting how AI-integrated e-HRM systems can enable real-time decision-making, foster continuous learning, and improve strategic alignment in resource-limited settings. By offering context-specific frameworks, it provides actionable strategies for enhancing responsiveness and competitiveness in developing technology markets.

Existing research on strategic agility and real-time decision-making in the technology industry underscores the transformative role of AI and e-HRM in enhancing organizational responsiveness, talent alignment, and data-informed leadership ( Zenjari, et al., 2024; Gautam, et al., 2025; Rahman, 2025). Several studies have investigated the effects of AI on specific HR functions like talent acquisition, performance management, and learning and development ( Kadirov et al., 2024, April; Paramita et al., 2024), while others have focused on how agile practices and digital innovation strategies support adaptability in dynamic tech environments ( Omowole, et al., 2024; Adhiatma et al., 2023). However, the literature often lacks a comprehensive view of how AI-integrated e-HRM systems holistically support strategic agility and real-time decision-making across different organizational settings.

Most existing studies center on developed economies and emphasize operational efficiencies (Al Samman, et al., 2024), overlooking the broader strategic impacts on long-term adaptability and workforce transformation. Moreover, limited attention has been given to how AI and e-HRM can align social and technical subsystems, as proposed by Socio-Technical Systems (STS) theory, to improve decision-making in technology firms. The absence of empirical data from developing economies further highlights a gap, especially where digital HR solutions are most needed to overcome institutional and infrastructural barriers. This study addresses these shortcomings by presenting a holistic framework that links AI-enabled e-HRM practices to strategic agility across both developed and developing contexts. It offers actionable insights for HR leaders seeking to strengthen organizational adaptability in increasingly volatile markets.

This review investigates how AI and e-HRM systems enhance strategic agility and real-time decision-making in the technology industry. It explores how AI-integrated e-HRM practices; such as predictive workforce analytics, AI-driven talent acquisition, AI-based learning and development (L&D), strategic scenario modeling, and digital employee experience (DEX) platforms, can help technology firms respond swiftly and effectively to rapidly evolving market conditions. Grounded in STS theory, the study also examines how aligning technical tools with human systems can drive holistic transformation, foster employee engagement, and promote innovation.

### Rationale for the review

The review holds significance for both academic and practical audiences. For researchers, it addresses a critical gap by presenting an integrated framework that connects AI-enabled e-HRM tools with strategic agility, particularly within the often-overlooked context of developing economies. For practitioners, especially HR and technology leaders, the study offers empirical evidence and strategic insights into building agile, data-informed HR systems that support real-time decision-making and organizational resilience. The findings have implications for shaping policy, building capabilities, and guiding digital transformation strategies that enhance competitiveness across both developed and developing technology markets.

The following are the objectives guiding this review:

1. To examine how AI and e-HRM systems contribute to strategic agility and real-time decision-making in the technology industry.
2. To assess the influence of AI-integrated e-HRM practices on the responsiveness and adaptability of technology firms.
3. To analyze the role of the STS theory in explaining the joint optimization of AI and e-HRM systems in enhancing organizational transformation, innovation, and employee engagement.
4. To identify the critical challenges faced by technology firms in adopting AI and e-HRM for strategic agility and data-driven decision-making.
5. To provide evidence-based recommendations for HR and technology leaders on how to effectively deploy AI-enabled e-HRM systems to enhance competitiveness, resilience, and policy development in the technology sector.
6. To explore the practical implications of implementing AI-enabled e-HRM systems, focusing on how their adoption influences HR workflows, leadership decision-making, workforce capability development, and the design of agile operating models within technology firms.

The research question “ *How are AI and e-HRM enhancing strategic agility and real-time decision-making in the technology industry?”,* forms the foundation of this review. It explores how digital innovations are reshaping the way technology firms respond to challenges in volatile, uncertain, complex, and ambiguous (VUCA) environments. The aim is to understand how AI and e-HRM systems support fast, data-driven decisions and enable firms to remain flexible, competitive, and adaptive in the face of rapid technological shifts.

To address this question, the review evaluates how AI-integrated e-HRM practices are being deployed in practice. It assesses how these tools enhance both technical systems and human capabilities, consistent with the principles of STS theory, to drive innovation, workforce engagement, and continuous learning.

By analyzing cases from both developed and developing economies, the review identifies the key enablers and barriers to successful AI and e-HRM adoption. It provides a comprehensive view of how digital HR transformation influences organizational agility and real-time responsiveness. The findings contribute to academic literature while offering actionable insights for HR professionals, technology leaders, and policymakers striving to build resilient, future-ready organizations.

## Materials and methods

This systematic review compiles and critically analyzes existing literature on how AI and e-HRM systems contribute to strategic agility and real-time decision-making in the technology sector. The review emphasized core thematic areas, including Predictive Workforce Analytics, AI-Driven Talent Acquisition, AI-Based Learning and Development, Strategic Scenario Modelling, and Digital Employee Experience Platforms. The primary aim is to identify the dominant AI tools and e-HRM functions being implemented, examine their effectiveness in enhancing organizational responsiveness, and evaluate challenges and ethical concerns surrounding their deployment. The review was conducted in accordance with the PRISMA 2020 guidelines for systematic reviews in management and information systems research as shown in Figure 2.

**Figure 1.**
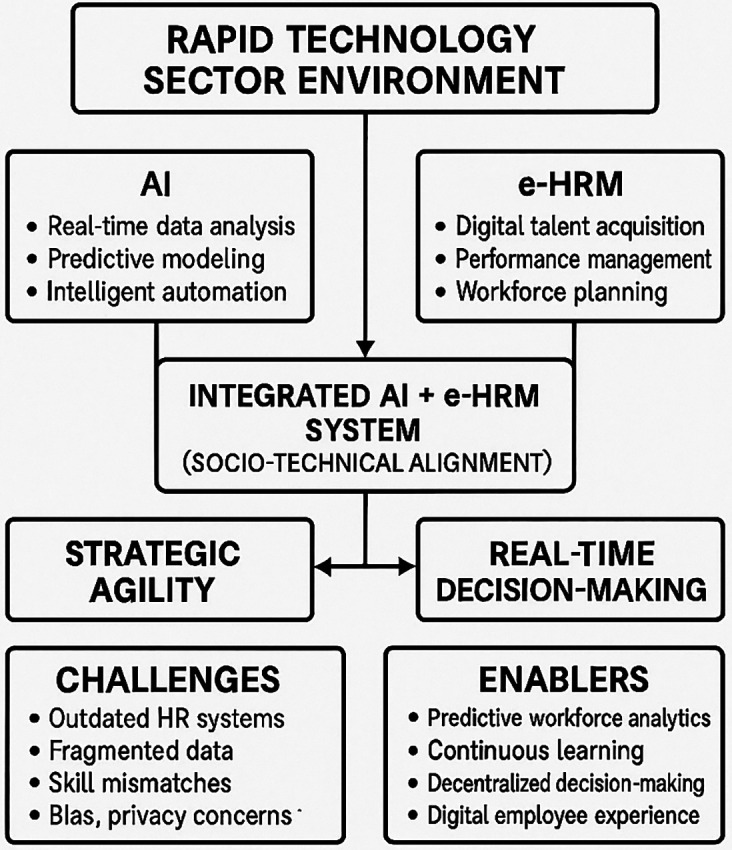
Rapid technology sector environment.

**Figure 2.**
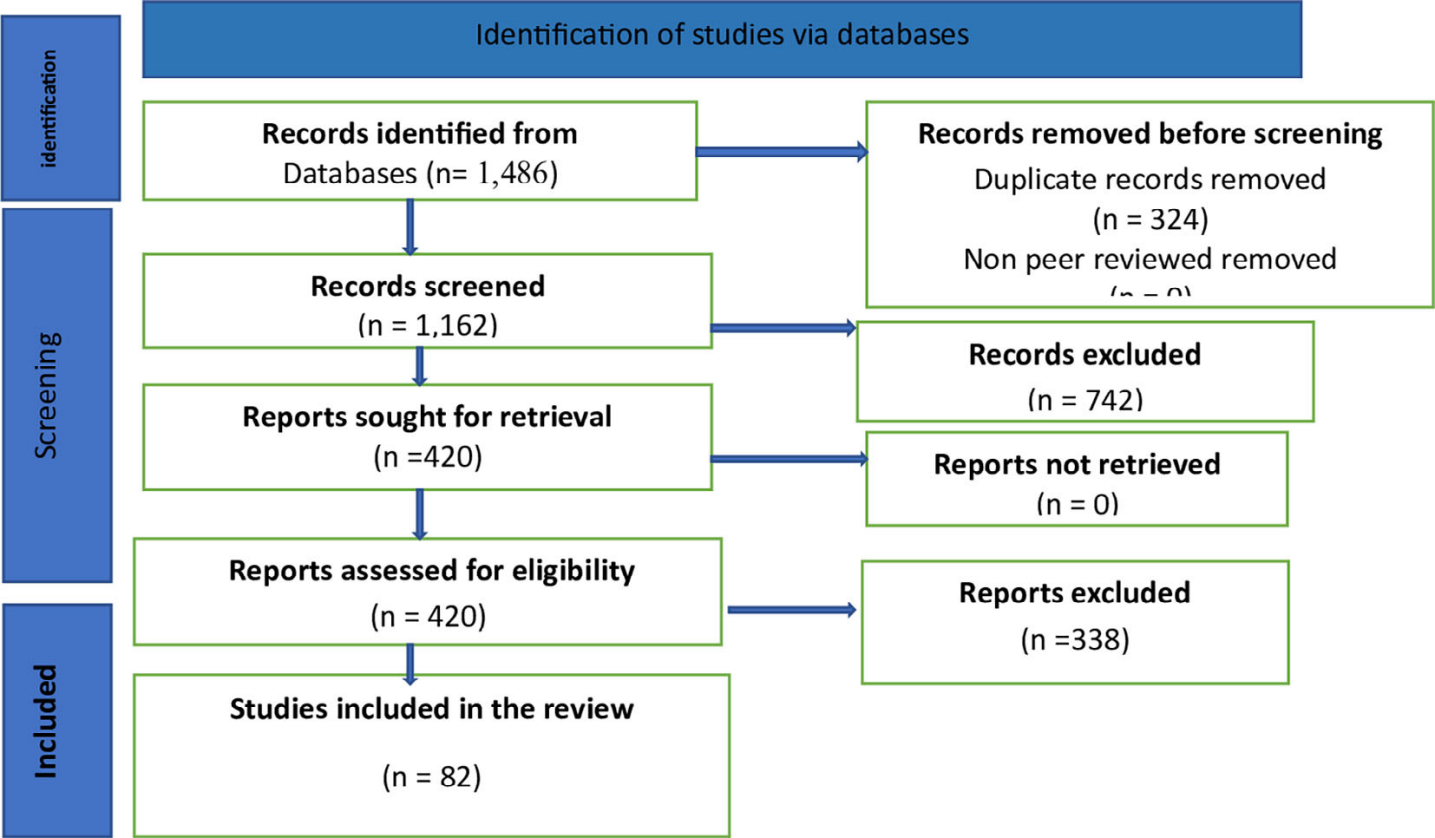
The PRISMA.

### Search methodology

The search strategy combined Boolean operators and key terms tailored to the research objectives. A comprehensive search was conducted using three major academic databases: Scopus, Web of Science, Google scholar and IEEE Xplore. Search terms were constructed using Boolean logic to combine keywords related to AI technologies (e.g., “machine learning,” “predictive analytics,” “intelligent systems”), e-HRM practices (e.g., “digital HRM,” “automated recruitment,” “e-performance management”), and organizational agility (e.g., “strategic agility,” “real-time decision-making,” “adaptive capability”). Truncation symbols (e.g., *) were employed to broaden the retrieval of word variants. The search was limited to English-language, peer-reviewed journal articles published between January 2015 and December 2025. The nomenclature and analysis scripts have been publicly archived at Tom Ongesa, N. (2025). Enhancing Strategic Agility and Real-Time Decision-Making in the Technology Sector: Exploring the Role of AI and e-HRM Systems – A Systematic Literature Review. Zenodo. https://doi.org/10.5281/zenodo.17378010 and are available under an open license.

### Criteria for selection

Two independent reviewers screened the records using structured inclusion and exclusion criteria. Discrepancies were resolved by consensus or third-party adjudication.

Title Examination: Duplicates were removed using Zotero software. Titles were evaluated to eliminate irrelevant studies, particularly those focused on non-technological industries or theoretical models lacking applied e-HRM or AI components.

Abstract Screening: Reviewers evaluated abstracts for relevance to AI/e-HRM integration and real-time strategy execution. Irrelevant studies, such as those focusing exclusively on HR training or traditional management models, were excluded.

Full-Text Review: Eligible studies underwent full-text review, with a focus on methodological transparency, alignment with the research focus, and clarity of strategic outcomes related to agility or decision-making.

### Criteria for inclusion and exclusion

**Inclusion criteria:**

1. Empirical studies, case studies, or reviews published between 2015–2025.
2. Studies analyzing the impact of AI or e-HRM tools on organizational agility, real-time analytics, decision-making speed, or strategic responsiveness.
3. Research addressing one or more of the five thematic areas (Predictive Workforce Analytics, AI-Driven Talent Acquisition, AI-Based Learning and Development, Strategic Scenario Modelling, Digital Employee Experience Platforms).
4. Research conducted within technology-driven industries or digital enterprises.
5. Articles offering quantitative outcomes, qualitative insights, or process frameworks on AI-driven HRM or strategic agility.

**Exclusion criteria:**

1. Non-peer-reviewed publications, editorials, white papers, and conference abstracts.
2. Studies unrelated to AI, HRM digitalization, or decision-making frameworks.
3. Articles limited to conceptual discussions without practical or measurable implications.
4. Research not contextualized within tech-based industries.
5. Literature confined to theoretical models without empirical validation or real-world application.

### Evaluation of bias

To assess the methodological robustness of included studies, researcher applied the Mixed Methods Appraisal Tool (MMAT) for empirical research. Each study was assessed on data collection integrity, transparency of AI/e-HRM implementation, and clarity in defining decision-making or agility metrics. The study also reviewed funding disclosures and potential conflicts of interest, especially in industry-sponsored studies or proprietary software evaluations.

### Synthesis methodologies

**Qualitative synthesis**

A qualitative and thematic synthesis approach was employed to analyze the collected studies. Each study was initially screened based on title and abstract, followed by full-text analysis for relevant articles. Data extraction sheets were developed to capture key findings, methodologies, sample contexts, and relevance to the thematic categories. The extracted data were then subjected to thematic analysis, guidelines, to identify recurring patterns, divergences, and conceptual advancements.

Thematic coding was used to categorize studies into functional clusters:

1. AI-enabled e-HRM applications (e.g., talent analytics, AI-powered recruitment, digital learning and development systems, Digital Employee Experience Platforms).
2. Strategic agility outcomes (e.g., scenario forecasting, real-time HR responsiveness, adaptive workforce strategies).
3. Technological enablers and barriers (e.g., cloud infrastructure, integration with ERP systems, data privacy concerns).

Emerging themes were organized under:

1. Application trends
2. Mechanisms of strategic impact
3. Implementation barriers

### Ethics

This review is based on open-access, secondary data sources and did not involve direct human subjects, thus exempting it from formal ethics board review. Nevertheless, the analysis acknowledges ongoing ethical debates in AI-e-HRM systems such as algorithmic bias, transparency, data surveillance, and implications for employee autonomy and digital labor rights.

### Research questions

What AI and e-HRM tools are most frequently used to support strategic agility and real-time decision-making in technology firms?

What measurable impact do these digital tools have on organizational responsiveness and HR adaptability?

What implementation barriers, ethical concerns, or contextual limitations affect the effectiveness of AI-driven HRM in real-world technology settings?

## Results

### Study identification and selection

A comprehensive search of the Scopus, Web of Science, Google Scholar and IEEE Xplore databases yielded 1,486 records. After the removal of 324 duplicates using Zotero, 1,162 unique titles and abstracts were screened. Based on predefined inclusion and exclusion criteria, 742 articles were excluded for irrelevance (e.g., non-tech sector, conceptual-only models, absence of AI or e-HRM focus).

The remaining 420 articles were assessed for full-text eligibility. Of these:

193 were excluded for insufficient detail on AI/e-HRM mechanisms

106 lacked measurable outcomes related to strategic agility or decision-making

39 failed quality appraisal standards (e.g., incomplete methodology, biased reporting)

Ultimately, 82 studies were included for qualitative synthesis. This is reflected in Table 1.

### Themes emerging from qualitative synthesis

1. AI-Powered Decision Tools in HRM
  Predictive analytics for workforce planning
  Machine learning–based talent acquisition systems
  NLP-powered sentiment and engagement monitoring
2. e-HRM Functional Transformations
  Automated performance management platforms
  Self-service HR portals with real-time dashboards
  Digital onboarding and learning environments
3. Strategic Agility Outcomes
  Enhanced scenario modeling and response time
  Agile workforce restructuring during crises (e.g., COVID-19, layoffs)
  Faster decision loops in HR operations and leadership support
4. Barriers to Implementation
  Data quality and system integration challenges
  Resistance to change from HR professionals

### Theoretical framework

This systematic literature review is grounded in Socio-Technical Systems (STS) Theory, introduced by Trist and Emery in 1951. STS Theory posits that organizations consist of two interdependent systems: the social system, which includes human relationships, culture, values, and psychological needs, and the technical system, which comprises tools, technologies, and procedures that drive operational goals ( Thomas, 2024; Zhang, et al., 2025). Optimal performance and employee satisfaction, according to the theory, are achieved when these systems are designed together, ensuring that technological efficiency does not come at the expense of human engagement or well-being ( Ing, 2024; Guest, et al., 2022; Zhang, et al., 2025).

STS Theory offers a relevant framework for analyzing how AI and e-HRM can enhance strategic agility and decision-making within the technology sector ( Roul, et al., 2024). As digital workplaces evolve, this dual focus on social and technical alignment becomes essential for organizations adapting to constant innovation and workforce transformation. AI-integrated e-HRM systems, such as predictive analytics and performance platforms, only generate value when they are interpretable, trustworthy, and embedded within human-centered processes ( Madancian & Taherdoost, 2023, October). For example, platforms like Adobe’s Check-in provide continuous feedback, aligning personal and organizational goals while supporting responsive and inclusive decision-making ( Pathirana, 2024).

Real-time data integration in AI-powered e-HRM tools enables faster, evidence-based decisions while preserving employee agency and fostering transparency. AI also supports personalized L&D programs by tailoring training to individual career goals and learning preferences, reinforcing the STS emphasis on contextual relevance and human motivation ( Zenjari et al., 2024; Kamal et al., 2024). In fast-paced industries like technology, AI-driven scenario modeling tools help firms explore strategic options ( Ji et al., 2024; Kaggwa et al., 2024). STS Theory stresses the importance of involving cross-functional teams in using these tools to ensure decisions are both technologically robust and socially grounded.

Overall, STS Theory guides the deployment of AI in e-HRM by advocating for a balanced evolution of technical tools and human-centered design. In technology-driven environments, where adaptability and talent are key to competitiveness, this integrated approach is crucial for sustainable strategic success ( Zenjari et al., 2024; Kamal et al., 2024).

### Literature review

To enhance strategic agility and real time decision-making in the technology sector through Artificial AI and e-HRM, there is need to embrace deliberate focus on high-impact areas that support Predictive Workforce Analytics, AI-Driven Talent Acquisition, AI-Based Learning and Development, Strategic Scenario Modeling, and Digital Employee Experience to enhance digital transformation, talent optimization, and strategic adaptability.

### Predictive workforce analytics for strategic agility and real-time decision-making in the technology sector

In the fast-changing technology industry, organizations must maintain strategic agility to navigate constant innovation, shifting markets, and increasing competition ( Ononiwu et al., 2024). Predictive workforce analytics, powered by AI, has become a critical component of e-HRM systems, providing advanced tools that support timely and informed talent decisions ( Roul et al., 2024). By leveraging both historical and real-time HR data, companies can forecast workforce trends, predict turnover risks, and identify future skill gaps. These insights allow decision-makers to align talent strategies with evolving business objectives, strengthening organizational responsiveness and operational flexibility ( Burnett & Lisk, 2021).

Rahaman and Bari (2024) emphasize that predictive analytics enhances long-term agility by enabling firms to anticipate talent shortages and future training needs. IBM’s integration of AI tools like Watson Analytics demonstrates this capability by tracking attrition risks, identifying high-potential employees, and supporting proactive retention efforts. This approach helps IBM channel talent toward innovation projects or reskilling pathways, ensuring adaptability in dynamic markets ( Singh, 2024). Accenture’s strategic use of predictive analytics further highlights its value by identifying obsolete roles and developing targeted reskilling programs ( Lakshmi et al., 2024). This not only preserves institutional knowledge but also builds critical skills aligned with the company’s digital transformation goals, reinforcing talent relevance in competitive service sectors ( George, 2024).

Other organizations across the Asia-Pacific region also demonstrate the benefits of predictive analytics. TCS reduced attrition by 20% in key roles by using AI to analyze feedback and turnover trends, launching customized engagement and mentorship programs that strengthened both retention and client service delivery ( Sharma & Rastogi, 2025; Verma et al., 2023). Smaller firms benefit as well; for example, a mid-sized cloud company in Singapore used AI-driven e-HRM tools to forecast hiring needs based on project pipelines and skill shortages ( Thakurta (2025)). This reduced recruitment delays and lowered dependence on contractors, improving project delivery and workforce readiness ( Radicic & Petković, 2023; Kraft et al., 2022). Predictive workforce analytics shifts talent management from a reactive process to a proactive, strategic capability, equipping technology firms with the foresight to meet future workforce challenges and sustain innovation, resilience, and performance in volatile environments ( Rahaman & Bari, 2024).

### AI-driven talent acquisition for strategic agility and real-time decision-making in the technology sector

AI-driven talent acquisition has become a transformative force in the technology industry, where speed, skill specificity, and innovation are critical for maintaining competitive advantage ( Hamadneh et al., 2024). Through AI-enabled e-HRM systems, technology firms can streamline recruitment by automating key processes such as candidate sourcing, screening, skill matching, and interview coordination ( Johnson et al., 2021). These advancements not only reduce hiring cycle times but also improve the alignment between candidate skills and job requirements, directly supporting strategic agility ( Pandey et al., 2023). Research by HAWRYSZ (2025) highlights that AI tools using natural language processing (NLP) and machine learning significantly improve quality-of-hire by predicting job fit and future performance while minimizing unconscious bias in early screening. This capability is especially valuable in the tech sector, where diverse talent and precise skill matching are essential for driving sustained innovation ( Madanchian, 2024; Somavarapu and Sharma, 2025).

Real-world applications illustrate these advantages. Google’s AI-powered applicant tracking system (ATS) utilizes pattern recognition and contextual analysis to assess candidate-job matches, predict hiring outcomes, and detect algorithmic biases to promote fairness ( Albassam, 2023; Shandy et al., 2025). Microsoft’s Azure-based recruitment platform further demonstrates the benefits of automation by managing early screenings and behavioral assessments, offering real-time insights to recruitment teams ( Valavanidis, 2023). This approach has led to significant reductions in time-to-hire while improving candidate engagement—both critical success factors in high-velocity environments ( Yadav et al., 2023). Infosys also exemplifies strategic AI integration through its platform “Nia,” which scans resumes, recommends learning pathways, and matches partially qualified applicants with future roles ( Learning et al., 2023; Andreu, 2024). This proactive model builds a long-term talent pipeline aligned with digital transformation goals while enhancing organizational agility.

Beyond efficiency, ethical AI design supports diversity and inclusion in recruitment. By anonymizing applications and applying objective algorithms, AI tools reduce the potential for gender and racial biases, fostering more inclusive work environments that benefit from varied perspectives ( Vivek, 2023; Emma, 2024b). This review confirms that AI-driven hiring advances strategic agility by enabling faster, data-informed talent decisions and allowing HR professionals to shift from transactional roles to strategic contributors. With repetitive tasks managed by AI, e-HRM systems position organizations to respond to evolving talent needs while strengthening long-term workforce preparedness.

### AI-based learning and development for strategic agility and real-time decision-making in the technology sector

In the fast-evolving technology sector, traditional training models no longer meet the demands of dynamic digital environments ( Carreno, 2024). AI-based L&D platforms have emerged as strategic tools to enhance agility, innovation, and workforce competitiveness ( Dixit & Jatav, 2024). Unlike static training programs, AI-enabled systems provide personalized, adaptive, and data-driven learning experiences. These platforms leverage algorithms to analyze employee performance, career goals, and prior learning to recommend timely, relevant content that promotes continuous upskilling aligned with organizational objectives ( Samuel et al., 2025; Nyathani, 2023). By supporting individual growth and facilitating strategic reskilling, AI-powered L&D strengthens the overall agility and responsiveness of the workforce ( Preechasin, 2024).

Amazon offers a compelling example of AI-driven internal learning. The company utilizes machine learning to create customized training pathways based on job roles and previous learning achievements. Warehouse employees, for instance, receive personalized courses in coding or cybersecurity if they express interest in transitioning to IT roles. This approach fosters internal mobility, reduces reliance on external hires, and ensures that the workforce evolves alongside Amazon’s broader digital transformation goals ( Liu, 2022; Sierszen & Drabek, 2024; Rane et al., 2024b). IBM’s “Your Learning” platform demonstrates another effective application of AI in workforce development. Powered by Watson, the platform analyzes employees’ roles, interests, and performance data to provide tailored training suggestions, including relevant certifications ( Pandita et al., 2025). Features like gamification and collaborative learning tools increase participation rates, and within its first year, a majority of IBM employees actively engaged with the platform, leading to more internal promotions and improved digital capabilities ( Awashreh & Hassiba, 2025).

Infosys also illustrates the strategic value of AI-enabled L&D through its LEX platform, which delivers microlearning tailored to real-time project demands and individual growth paths. The system curates content that keeps employees ahead of client expectations, improving both project outcomes and delivery speed ( Aithal & Rao, 2024). This approach resulted in higher employee satisfaction with training and stronger client feedback on service quality ( Agrawal, 2022). Beyond enhancing personal development, AI-based L&D platforms contribute to strategic workforce planning by analyzing aggregated learning data to forecast skill gaps and launch targeted training initiatives ( Yanamala, 2024). This is particularly valuable in the tech industry, where fast-paced advancements in AI, blockchain, and quantum computing continuously reshape job requirements ( Kaal, 2024). Organizations that embrace AI-driven L&D systems are better equipped to anticipate these shifts and remain competitive in global markets ( Nyathani, 2023).

AI-enabled learning is fundamentally transforming talent development in the technology sector by offering scalable, strategic, and highly targeted educational experiences. These platforms not only close critical skill gaps but also increase employee engagement and strengthen organizational agility. For firms focused on sustaining innovation and responsiveness in volatile environments, investing in AI-powered L&D has shifted from being an option to a necessity for long-term success.

### Strategic scenario modeling and strategic agility for real-time decision-making in the technology sector

AI-powered strategic scenario modeling is reshaping how technology firms anticipate change and make agile, informed decisions ( Nweke & Adelusi, 2025). By combining predictive algorithms with historical HR data, AI-driven e-HRM platforms enable organizations to simulate multiple workforce scenarios. These simulations help decision-makers proactively assess potential outcomes under diverse market conditions and organizational changes ( Pandey et al., 2023, 2024). This transformation allows companies to shift from reactive decision-making to proactive, forward-looking strategies that align closely with shifting business needs and evolving market demands.

AI-enabled modeling analyzes the potential impacts of factors such as labor market trends, regulatory shifts, and technological disruptions on workforce structure, performance, and engagement ( Patil, 2024). These simulations allow organizations to experiment with proposed policy changes, workforce restructuring, or talent investments in a risk-free environment before committing to full-scale implementation ( George, 2024). For example, a technology firm can use AI modeling to predict how introducing a hybrid work model might affect employee engagement or turnover, helping leaders make more confident, evidence-based decisions ( Kumari & Yelkar, 2022; Mahajan et al., 2024).

SAP’s Success Factors platform provides a notable example of predictive analytics in action within e-HRM. It allows HR leaders to simulate workforce decisions like outsourcing, relocation, or upskilling by integrating internal employee data with external labor market trends ( Dutta et al., 2025). During the COVID-19 pandemic, many of SAP’s clients used the platform to evaluate staffing flexibility and remote work strategies, enabling faster, data-driven responses to unprecedented disruptions ( Rang & Sjöstrand, 2021). Cisco Systems applied similar modeling capabilities during its transition to a subscription-based business model ( Pandey et al., 2023). Using AI-driven workforce simulations, Cisco identified future talent needs and skill gaps, facilitating targeted reskilling efforts that supported continuity throughout its business transformation ( Quimba et al., 2024). These proactive initiatives minimized disruption and enabled smoother execution of strategic shifts.

Evidence from Deloitte’s 2020 Global Human Capital Trends report underscores the value of scenario modeling, particularly during economic shocks. Organizations that had previously modeled responses to potential talent shortages or supply chain disruptions demonstrated greater resilience and adaptability during the pandemic by implementing well-developed contingency plans ( Schwartz, 2021; Ponce & Lustig, 2023). This reinforces the role of AI-integrated scenario modeling in strengthening organizational preparedness, agility, and long-term resilience.

Beyond immediate crisis management, scenario modeling plays a critical role in strategic workforce development ( Berman et al., 2022). Simulating the impact of automation on various job roles allows firms to plan proactively for reskilling, targeted recruitment, and succession planning ( Praba et al., 2025; Samuel et al., 2025). These predictive capabilities ensure that talent strategies remain aligned with evolving business models and technological advancements. Recent studies confirm that embedding AI-powered scenario modeling within e-HRM platforms has become essential for technology firms navigating market complexity and rapid innovation cycles ( Maheswari et al., 2024; Mahade et al., 2025). By forecasting how strategic choices influence workforce outcomes, organizations can enhance agility, resilience, and strategic alignment with future goals. As market disruptions accelerate, AI-driven scenario modeling will remain a cornerstone of future-ready, competitive HR strategies ( Paul, 2024; Emma, 2024c).

### Digital employee experience platforms for strategic agility and real-time decision-making in the tech industry

In the technology sector, where innovation and agility drive competitiveness, the digital employee experience (DEX) has become central to sustaining organizational performance. AI-powered e-HRM systems are transforming DEX by delivering intelligent, responsive, and data-driven platforms that elevate employee engagement, reduce administrative friction, and enhance talent retention ( Pandey et al., 2023, 2024). These systems tailor employee interactions by adapting interfaces to individual roles, preferences, and behaviors, providing personalized digital experiences that foster greater alignment with employee needs ( Eswaran & Eswaran, 2025; Priyanka et al., 2024). Common features include AI-driven chatbots offering 24/7 HR support, predictive engagement monitoring tools, and streamlined self-service portals for routine tasks ( Chadha, 2024; Satpathy et al., 2024). For technology firms where responsiveness underpins competitiveness, these capabilities are increasingly indispensable.

Salesforce exemplifies this AI-driven approach through its Einstein AI platform, which powers HR chatbots that deliver immediate responses to employee questions regarding company policies, benefits, and workplace procedures ( Bandella, 2025). These chatbots utilize machine learning to improve accuracy and relevance based on user interaction over time ( Parmar, 2023). Einstein AI also personalizes content delivery, ensuring employees receive updates and tools specifically tailored to their roles and responsibilities. This targeted approach fosters employee engagement and strengthens alignment between individual performance and strategic business objectives ( Kaliuta, 2023; Stefaniak, 2023). Microsoft’s Viva platform similarly integrates DEX into daily workflows by embedding learning, communication, and wellbeing features within Microsoft Teams. Using AI analytics, Viva monitors collaboration patterns and generates personalized recommendations to enhance work-life balance and professional growth ( Ekandjo, 2024; Ekandjo et al., 2023). This capability strengthens resilience, particularly in hybrid work environments, helping maintain consistent performance and engagement.

Research further highlights the significant impact of AI-enhanced DEX on employee retention and engagement. Digital tools featuring sentiment analysis and continuous feedback loops enable early detection of disengagement trends, allowing HR teams to implement timely interventions ( Ristola, 2024; Kafey, 2025). AI models analyze behavioral patterns to identify employees at risk of turnover, prompting tailored strategies that improve satisfaction and foster long-term commitment ( Omose & Ikuyinminu, 2024). IBM’s use of Watson AI illustrates the power of personalization in employee development. Watson provides employees with individualized career guidance and learning suggestions aligned with their aspirations and skillsets, reinforcing satisfaction, professional growth, and career alignment in knowledge-driven sectors ( Chakraborty & Majumder, 2024; Bashynska et al., 2023).

Beyond enhancing immediate work experiences, advanced DEX platforms contribute strategic value by preserving institutional knowledge, accelerating onboarding processes, and supporting performance consistency in project-based and fast-paced environments ( Surve et al., 2023). These systems also sustain strong organizational cultures in hybrid work models by fostering ongoing digital engagement, collaboration, and cohesive communication ( Yang, 2024; Hussain, 2024; Al-Shuwaikhat, 2024). This holistic support ensures that both operational efficiency and cultural continuity are maintained across diverse working arrangements.

AI-enabled DEX platforms have become essential assets for achieving strategic agility in the technology sector. By providing personalized employee interactions, delivering predictive insights, and enabling seamless engagement, these platforms empower organizations to retain talent, boost morale, and sustain operational continuity. These advantages are critical in navigating the demands of a dynamic, innovation-driven industry.

## Discussion of literature review

**Figure 3.**
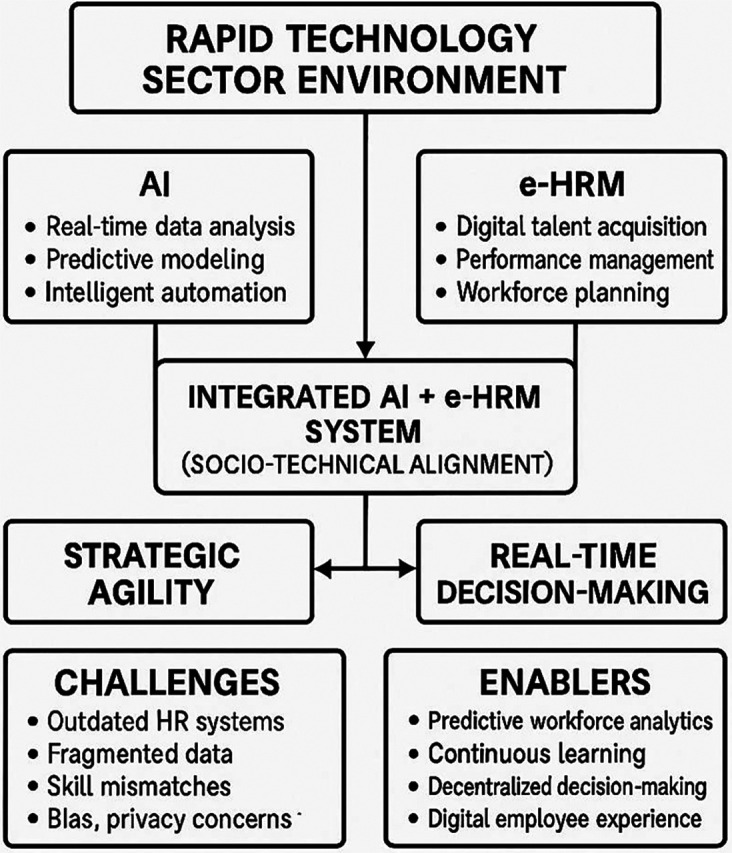
Conceptual framework.

The literature reviewed is critically analyzed so as to identify gaps, contradictions, and areas where further research is needed as shown if Figure 3 and subsequent tables.

**Table 1.** Demographic and sectoral characteristics of included studies.

Geographic Spread	Percentage
North America	(28%)
Europe	(25%)
Asia	(18%)
Africa	(9%)
Multi-regional studies	(20%)
Industry Contexts	
Software and IT services	(35%)
Fintech and telecommunications	(24%)
Digital HR consultancies	(17%)
Tech-driven healthcare and education sectors	(12%)
Cross-sector digital transformation projects	(12%)

**Table 2.** Critical review of literature on predictive workforce analytics for strategic agility and real-time decision making in the technology sector.

Theme	Key Findings	Challenges/Gaps Identified
Predictive Analytics in Talent Management	AI-driven tools can forecast attrition, identify high-potential talent, and inform leadership development ( Rahaman & Bari, 2024).	Limited research on ethical concerns such as data bias, privacy, and employee trust in AI-driven HR systems.
Strategic Workforce Planning	Organizations like IBM and TCS use predictive analytics to align talent supply with strategic goals, ensuring business continuity and agility ( Singh, 2024; Sharma & Rastogi, 2025; Verma et al., 2023).	Under-representation of SMEs and firms from developing economies; current studies mostly focus on resource-rich multinationals.
Reskilling and Skills Forecasting	Companies like Accenture use AI to predict skill obsolescence and implement digital training strategies ( Burnett & Lisk, 2021).	Lack of longitudinal studies assessing the sustained impact of predictive analytics on long-term workforce agility and innovation performance.
Scalable Adoption in Smaller Firms	Evidence from mid-sized firms ( Thakurta, 2025) shows AI-based e-HRM tools can improve efficiency and reduce dependency on contractors.	Limited examination of infrastructure readiness and digital maturity in less digitized or low-resource environments; barriers to adoption in developing regions not well explored.
Strategic Agility through Proactive HR	Predictive analytics enables a shift from reactive to proactive HR, enhancing responsiveness to market changes and technological volatility ( Rahaman & Bari, 2024).	Insufficient research on cross-cultural or contextual influences on AI-e-HRM effectiveness, especially in varying organizational cultures and regulatory environments.
Integration of Socio-Technical Systems (STS) Theory	STS approach suggests the combination of AI and HR systems supports agility and innovation.	Few empirical studies explicitly apply STS theory to measure the holistic impact of integrating AI tools with human systems in diverse organizational settings.

**Table 3.** Critical analysis of literature on AI-driven talent acquisition for strategic agility in the technology sector.

Thematic Area	Strengths in Literature	Identified Gaps/Challenges	Further Research Needed
**Recruitment Efficiency**	AI tools reduce hiring time, automate screening, and match skills effectively ( Albassam, 2023).	Focus mainly on large tech firms; limited insights into SMEs' access to such tools.	Explore scalability and effectiveness of AI-driven hiring in small and mid-sized tech companies.
**Bias and Diversity**	AI can mitigate human bias and promote fair hiring ( Vivek, 2023).	Ethical contradictions persist due to algorithmic opacity and biased training data.	Assess AI tools' performance in real-world diversity outcomes across demographic and cultural contexts.
			
**Post-Hire Outcomes**	Case studies show improved initial matching ( Learning et al., 2023; Andreu, 2024).	Limited evidence on post-hire performance, retention, and long-term adaptability.	Longitudinal studies on the long-term effectiveness of AI hires in dynamic tech environments.
**Candidate Experience and Trust**	AI tools offer quicker response times and structured processes ( Johnson et al., 2021).	Lack of research on candidates' perception of AI-driven processes, including trust and fairness.	Study psychological and behavioral impacts of AI-based hiring on applicants' experience and trust.
**Global and Legal Applicability**	Leading tech firms show global application success (Google, Microsoft, Infosys cases).	Limited research on alignment with diverse labor laws and ethical standards globally.	Evaluate AI hiring systems' compatibility with legal and regulatory frameworks across global regions.

**Table 4.** AI-based learning and development platforms enhancing strategic agility in the technology sector.

Company	AI-Based L&D Platform	Key Features	Strategic Benefits	Reported Outcomes
Amazon	Internal ML-Powered Platform	Personalized training paths based on role, skills, and career goals	Supports internal mobility, reduces external hiring needs	Transition support to IT roles; aligns training with digital transformation goals
IBM	Your Learning (Watson AI)	Real-time course recommendations, gamification, and performance-based analytics	Encourages self-directed learning and strategic skill acquisition	Over 90% engagement; uplift in promotions and digital skills acquisition
Infosys	LEX Platform	Microlearning modules curated based on project demands and career paths	Aligns training with real-time delivery needs; enhances workforce flexibility	40% increase in training satisfaction; improved project delivery feedback

**Table 5.** AI-driven strategic scenario modelling in the technology sector: Applications, benefits, and research gaps.

Aspect	Description
**Core Concept**	Use of AI-powered e-HRM systems for simulating strategic HR decisions under various market, economic, and organizational scenarios.
**Key Technologies**	Predictive analytics, machine learning algorithms, real-time workforce simulations, external data integration (e.g. market trends, policy changes).
**Primary Benefits**	Enhances strategic agility, supports proactive HR planning, forecasts workforce needs, minimizes business risks, and optimizes talent investments.
**Leading Examples**	- SAP SuccessFactors: Simulates impacts of staffing decisions under different scenarios ( Dutta et al., 2025). - Cisco Systems: Forecasts reskilling needs linked to strategic pivots ( Pandey et al., 2023).
**Empirical Strengths**	Shows improved organizational responsiveness and workforce resilience during disruptions like COVID-19 ( Rang & Sjöstrand, 2021).
**Research Gaps**	- Limited data on adoption in small and mid-sized firms. - Lack of longitudinal and empirical validation of outcomes. - Scarcity of peer-reviewed studies.
**Contradictions Identified**	Predictive accuracy may decline in volatile environments; real-time data integration remains challenging.
**Further Research Needs**	- Assess effectiveness across varied organizational sizes. - Explore ethical concerns (e.g., algorithmic bias). - Examine employee perceptions and trust.

**Table 6.** Critical analysis of AI-powered digital employee experience platforms in the technology sector.

Aspect	Key Insights	Identified Gaps and Challenges
**Strategic Value of DEX Platforms**	AI-driven DEX platforms like Salesforce Einstein and Microsoft Viva enhance engagement, wellbeing, and strategic agility ( Bandella, 2025; Ekandjo, 2024; Ekandjo et al., 2023).	Over-reliance on case studies from large corporations; lack of research on effectiveness in SMEs and start-ups.
**Personalization and Predictive Insights**	Real-time analytics and behavioral data allow tailored HR services and early disengagement detection ( Eswaran & Eswaran, 2025; Priyanka et al., 2024).	Longitudinal evidence on the sustained impact of predictive DEX tools is limited.
**Examples of Implementation**	IBM Watson and Microsoft Viva use AI for personalized development and wellbeing recommendations, improving retention and morale ( Ekandjo, 2024; Ekandjo et al., 2023; Chakraborty & Majumder, 2024; Bashynska et al., 2023).	Empirical studies needed to verify the replicability of success across different regions, firm sizes, and digital maturity levels.
**Employee Trust and Ethics**	AI tools offer efficiencies but raise concerns about surveillance, data privacy, and algorithmic bias.	Need for studies on employee trust in AI, ethical design of digital HR tools, and governance frameworks to ensure transparency and accountability.
**Cultural and Generational Factors**	Digital platforms claim universal benefit through customization, but their usability may vary based on demographic preferences and tech literacy.	Lack of research on how generational or cultural diversity affects the adoption, effectiveness, and acceptance of AI-enhanced DEX systems.
**Future Research Needs**	There is consensus that AI-powered DEX supports strategic continuity and human capital development in tech firms.	Further research needed on cross-cultural impact, algorithmic fairness, user autonomy, psychological safety, and generalizability across non-tech or hybrid work environments.

**Table 7.** Comparative insights on predictive workforce analytics in the technology sector.

Source/Study	Focus Area	Findings from Earlier Studies	Findings from Recent Corporate Implementations	Key Similarities	Key Differences
Ononiwu et al., 2024; Roul et al., 2024; Burnett & Lisk, 2021	Predictive Modelling in HR	Emphasized predictive analytics as a driver for aligning HR with long-term business objectives and anticipating workforce shifts.	AI-driven predictive models used by firms like IBM and Accenture to forecast skill demands, attrition risks, and future workforce needs ( Singh, 2024; Lakshmi et al., 2024).	Both emphasize predictive modelling as foundational to strategic HR alignment.	Recent models integrate real-time AI forecasting for faster, continuous workforce strategy refinement ( Subrahmanyam, 2025; Gowrishankkar et al., 2025).
Singh, 2024; Lakshmi et al., 2024	Analytics-Enhanced HR Systems	Demonstrated that integrated analytics improves forecasting accuracy for workforce planning, including identification of skills gaps.	IBM and Accenture leverage AI for predicting employee behavior, job-role compatibility, and future training needs ( Gowrishankkar et al., 2025; Subrahmanyam, 2025).	Both confirm analytics improves decision-making precision in HR planning.	Contemporary models use AI to incorporate behavioral prediction, beyond earlier skills-based forecasting ( Subrahmanyam, 2025).
Zong & Guan, 2024; Sharma & Rastogi, 2025	Use of Analytics in Large Corporations	Focused predominantly on large, multinational firms with sophisticated data infrastructures, prioritizing cost reduction and operational efficiency.	Mid-sized technology firms in Asia increasingly adopting predictive hiring tools via cloud-based HR systems to enhance resource allocation agility (Lee & Low, 2019).	Predictive analytics viewed as essential for improving HR operational efficiency.	Recent adoption by mid-sized firms signals broader accessibility through modular, cloud-based solutions (Lee & Low, 2019).
Smit et al., 2025	AI Use in Mid-Tier Companies in Asia	Earlier studies overlooked smaller technology firms, focusing mostly on large-scale enterprises with dedicated HR analytics functions.	Demonstrated successful adoption of AI-based predictive analytics in mid-sized technology firms, improving hiring timelines and workforce agility (Lee & Low, 2019).	Shared recognition of predictive analytics improving recruitment efficiency.	Shift in focus from predominantly large firms to regional and mid-sized enterprises leveraging accessible AI-powered tools.
Saleh et al., 2024; Adewusi et al., 2024; Nyoni, 2025	Predictive Workforce Planning & Engagement	Initial focus was on descriptive analytics and cost-efficiency metrics, with limited attention to engagement, resilience, or future innovation readiness.	Predictive analytics now used to anticipate skills obsolescence, improve engagement strategies, and maintain innovation continuity in fast-changing environments ( Subrahmanyam, 2025).	Both recognize predictive analytics supports proactive workforce planning in technology firms.	Recent literature emphasizes predictive analytics as an enabler of sustained innovation, employee engagement, and resilience ( Nyoni, 2025).

**Table 8.** Comparative insights on AI-driven talent acquisition practices in the technology sector.

Study/Source	Focus Area	Findings from Earlier Studies	Findings from Recent Corporate Implementations	Key Similarities	Key Differences
Hukkeri & Pol, 2025; Bhushan, 2024	AI in Recruitment Screening and Bias Reduction	Identified AI’s potential to reduce recruitment bias, automate CV screening, and enhance hiring quality using ML and NLP.	Microsoft and Infosys employ real-time analytics, behavioral assessments, and AI-led talent pooling systems ( Valavanidis, 2023; Andreu, 2024; Learning et al., 2023).	Both emphasize improved hiring efficiency and better job-fit outcomes.	Recent AI systems facilitate real-time candidate assessments and dynamic pooling integrated into broader HR strategies ( Hamadneh et al., 2024).
Hukkeri & Pol, 2025; Bhushan, 2024	Skill-Based Hiring Accuracy	Highlighted AI’s contribution to improving candidate-job fit using data-driven matching based on qualifications and experience.	Google deploys AI-driven talent mapping and predictive modelling to align hiring with future organizational skill requirements ( Albassam, 2023; Shandy et al., 2025).	Emphasis on skill-precision in candidate selection.	Recent AI focuses on predicting future skill needs for strategic workforce alignment, not just immediate job role matching ( Valavanidis, 2023).
Bhushan, 2024	Ethics and Strategic Decision Support	Addressed algorithmic fairness, risk of embedded bias, and the role of AI in supporting strategic HR decision-making.	Microsoft integrates ethical auditing, fairness metrics, and diversity-focused recruitment features in its AI hiring systems (Shandy et al., 2025; Albassam, 2023).	Focus on ethical deployment of AI in recruitment.	Contemporary platforms incorporate embedded fairness auditing and inclusion metrics as standard features ( Valavanidis, 2023; Hamadneh et al., 2024).
Learning et al., 2023; Andreu, 2024	AI-Enabled Adaptive Hiring Pathways	Earlier research offered limited exploration of AI supporting development paths for partially qualified candidates.	Infosys uses AI to recommend targeted skill-building opportunities for candidates, linking hiring with L&D to build strategic talent ecosystems ( Learning et al., 2023).	Common focus on improving overall talent pipeline quality.	New systems connect recruitment with candidate development, supporting adaptive workforce readiness ( Andreu, 2024; Hamadneh et al., 2024).

**Table 9.** Comparative analysis of AI-driven learning and development platforms in technology firms.

Study/Source	Focus Area	Findings from Previous Studies	Findings from Recent Corporate Experiences	Key Similarities	Key Differences
Liu, 2022; Sierszen & Drabek, 2024	Personalized Learning	Identified the drawbacks of static, one-size-fits-all training; advocated for personalized, adaptive learning paths.	Amazon’s ML-driven learning and IBM’s Watson-powered “Your Learning” platforms provide real-time, role-based, individualized learning content ( Carreno, 2024).	Both support adaptive, personalized learning to improve engagement and skills.	Corporate platforms apply predictive modelling to forecast future skill needs, exceeding early concepts of static personalization ( Bobitan et al., 2024).
Dixit & Jatav, 2024; Preechasin, 2024; Samuel et al., 2025; Nyathani, 2023	Data-Driven L&D	Highlighted using employee data to align learning content with roles and organizational goals.	Infosys’s LEX platform dynamically integrates learning journeys with real-time project needs and feedback mechanisms ( Aithal & Rao, 2024).	Both emphasize using data for more targeted and relevant learning.	Newer systems incorporate continuous feedback loops and alignment with strategic business outcomes beyond static skill alignment ( Lokesh et al., 2024).
Bobitan et al., 2024; Lokesh et al., 2024	Efficiency in Upskilling	Stressed digital tools for faster upskilling but lacked focus on behavioral engagement.	Infosys and IBM platforms incorporate gamification, behavioral insights, and social features to enhance retention and engagement ( Pandita et al., 2025).	Common goal to improve upskilling access and speed.	Contemporary platforms add motivational and engagement features like gamification and social learning, absent in earlier frameworks ( Awashreh & Hassiba, 2025).
Samuel et al., 2025; Nyathani, 2023	Strategic Workforce Agility	Limited or absent emphasis on measurable strategic workforce agility or ROI tracking.	IBM and Infosys platforms track learning ROI, link training to internal promotions, and forecast organizational skill requirements ( Aithal & Rao, 2024).	Both view learning as a key to improved workforce performance.	Recent systems offer predictive analytics, business KPI alignment, and clear metrics like promotion rates—features missing in prior research.

**Table 10.** Comparative strategic scenario modeling in technology firms.

Source/Study	Focus Area	Findings from Earlier Studies	Findings from Recent Corporate Implementations	Key Similarities	Key Differences
Hao et al., 2024; Spaniol & Rowland, 2023; Pandey et al., 2023	Data-Driven HR Strategies	Emphasized predictive analytics for workforce planning with theoretical grounding, but limited empirical validation.	SAP SuccessFactors uses AI-driven scenario simulations for hybrid work, reskilling, and organizational transformation ( Dutta et al., 2025).	Both emphasize predictive analytics for HR strategic decision-making.	Recent models use real-time simulations tied to changing strategic business contexts, going beyond theoretical validation ( Quimba et al., 2024).
Wang & Zheng, 2024	Static Scenario Planning	Described HR scenario planning as sporadic, infrequent, and focused solely on internal HR metrics.	Cisco employs continuous, AI-enhanced modelling integrated with digital transformation strategies ( Mahajan et al., 2024).	Both aim to align HR planning with broader organizational needs.	Modern practices apply continuous simulation aligned with technological change, diverging from earlier isolated scenario exercises ( Pandey et al., 2023).
Dutta et al., 2025; Rang & Sjöstrand, 2021	Scenario Simulations in Uncertain Environments	Earlier models were mostly internally focused, lacking adaptability to external crises.	SAP used external, real-time data during the pandemic for workforce scenario modelling, enhancing responsiveness ( Quimba et al., 2024).	Both stress predictive modelling for preparedness.	Contemporary models incorporate external datasets for crisis readiness, unlike earlier inward-looking approaches.
Schwartz, 2021; Ponce & Lustig, 2023	AI-Enabled Strategic Resilience	Limited focus on empirical validation of AI’s contribution to organizational resilience.	Companies using AI-powered scenario planning adapted faster during crises like COVID-19, validating its role in resilience ( Nweke & Adelusi, 2025).	Both recognize forecasting’s importance for organizational strength.	Recent evidence demonstrates empirical agility improvements, whereas early research was theoretical or lacked real-world examples.

**Table 11.** Comparative analysis of AI-powered digital employee experience platforms in the technology sector.

Study/Source	Focus Area	Findings from Previous Studies	Findings from Current Use Cases	Key Similarities	Key Differences
Pandey et al., 2023, 2024; Biswas et al., 2024	e-HRM Platforms and Engagement	Highlighted AI’s potential for automating HR tasks and introducing predictive engagement mechanisms.	Salesforce, IBM, and Microsoft leverage AI chatbots and interactive platforms delivering real-time feedback, tailored support, and personalization.	Both highlight automation and predictive analytics in HR workflows.	Recent platforms emphasize strategic agility, real-time adaptation, and user-centric experiences with integrated feedback loops.
Parmar, 2023; Chakraborty & Majumder, 2024	Personalized Talent Development	Identified AI’s role in designing career progression and skill development pathways.	IBM’s Watson provides dynamic, real-time career coaching, adapting to employee behaviors and interests ( Bashynska et al., 2023).	Both stress personalization for employee development.	Contemporary tools offer adaptive pathways using real-time employee data, exceeding earlier static career planning approaches.
Soomro, 2020	Predictive HR Analytics and Retention	Suggested that real-time sentiment analysis could prevent disengagement and reduce attrition.	Microsoft Viva and Salesforce integrate predictive analytics directly within collaboration tools to provide preemptive engagement interventions.	Agreement on sentiment analysis’ role in engagement.	Current platforms integrate predictive features into everyday workflows (e.g., Microsoft Teams), unlike earlier detached analytical models.
Ekandjo, 2024; Zhang & Chen, 2024	AI in Wellbeing and Performance Management	Proposed combining wellbeing metrics with productivity tracking to improve strategic HR planning.	Microsoft Viva provides real-time suggestions for work-life balance, productivity enhancement, and employee wellbeing, embedded within work tools.	Both advocate linking wellbeing with performance.	Modern platforms translate wellbeing recommendations into immediate actions embedded in employee routines, improving effectiveness.

**Table 12.** Alignment and challenges of predictive analytics application with Socio-Technical Systems (STS) theory in the technology sector.

Study	Predictive Analytics Application	Strategic Outcome	STS Alignment
Pandey et al., 2023; Ononiwu et al., 2024	AI tools forecast attrition, identify high-potential employees	Improved talent retention and leadership development	Enhances human-centric planning by providing interpretable AI outputs to HR professionals
Burnett & Lisk, 2021; Nwoke, 2025	Predicts skills obsolescence, guides digital reskilling efforts	Future-ready workforce aligned with evolving service needs	Aligns learning and development strategies with both employee aspirations and technological trajectories
Thomas, 2024; Leal-Rodríguez et al., 2023	Predicts exit risk using surveys and performance data	Reduced attrition and improved business continuity	Integrates employee feedback and social insights into predictive HR decision-making processes
Okon et al., 2024; Ivchyk, 2024	Forecasts hiring needs based on project pipelines	Efficient hiring processes and reduced contractor reliance	STS model supports scalability by combining technical forecasts with leadership-driven workforce planning
Sasmal, 2024; Zong & Guan, 2024; Ramya et al., 2024	Anticipates training needs and talent shortages	Long-term agility through proactive workforce development	Joint optimization of organizational planning with technical predictive tools to maintain agility

**Table 13.** Alignment between STS theory and AI-driven talent acquisition in the technology sector.

Aspect	STS Theory Perspective	AI-Driven Talent Acquisition Application	Challenges
**Joint Optimisation**	Emphasises simultaneous design of technical systems and human work structures.	AI platforms like Infosys Nia automate screening while HR focuses on engagement and fit.	Risk of focusing too heavily on automation, sidelining human intuition and empathy in hiring.
**Human Augmentation**	Promotes technology as a tool to enhance—not replace—human roles.	AI enables HR teams to spend more time on strategic tasks like cultural fit and candidate experience.	Inadequate training or overdependence may reduce human critical thinking in final hiring decisions.
**Transparency & Fairness**	Requires systems to be understandable and just.	Platforms like Google’s qDroid embed fairness audits in candidate evaluation algorithms.	Black-box AI models can create opacity, leading to mistrust and unintentional bias.
**Organisational Agility**	Supports rapid adaptation through flexible socio-technical design.	Real-time AI insights enable swift hiring decisions aligned with shifting business needs.	Over-reliance on predictive models may not account for emerging or unconventional candidate profiles.
**Ethical and Cultural Sensitivity**	Encourages technology to reflect ethical standards and organisational values.	AI tools can be calibrated to detect discriminatory patterns and align hiring with inclusive practices.	Risk of cultural insensitivity or ethical lapses if algorithms are not regularly updated and reviewed.
**Continuous Calibration**	Advocates for ongoing alignment between social and technical components.	Feedback loops from HR and candidates guide AI model improvements and contextual adaptability.	Difficulty in maintaining alignment due to evolving job markets and organizational change.

**Table 14.** Alignment between STS theory and AI-based learning and development in the technology sector.

Aspect	Alignment with STS Theory	Challenges in Alignment
**Personalisation**	AI platforms tailor learning based on performance, roles, and aspirations, supporting human needs and enhancing motivation.	Over-personalisation may limit exposure to diverse knowledge and reduce informal learning opportunities.
**Human-Technology Integration**	Technology enhances workforce adaptability while maintaining employee agency, engagement, and development—key STS goals.	Opaque algorithms can lead to mistrust if employees cannot understand or validate AI-driven decisions.
**Strategic Agility**	AI-based L&D supports rapid skill development aligned with business goals, enhancing organisational responsiveness and agility.	Achieving real-time agility requires robust data infrastructure and continuous model training, which may strain resources.
**Workforce Engagement**	Systems like IBM’s Watson and Infosys LEX promote self-directed learning and social collaboration, reinforcing the STS social system.	Excessive focus on technical outcomes may neglect social learning environments and team-based collaboration.
**Decision-Making Support**	AI provides real-time, evidence-based insights, enabling faster and more informed HR and strategic decisions, in line with STS co-optimisation.	Lack of transparency in AI recommendations may reduce the perceived fairness and inclusivity of decision-making processes.
**Scalability and Contextual Fit**	Platforms can be scaled across global teams while being customised to individual contexts—balancing STS’s emphasis on flexibility and relevance.	Contextualising content for diverse workforces at scale demands high investment and sophisticated data governance to avoid bias and ensure inclusivity.

**Table 15.** Alignment between STS theory and strategic scenario modelling and strategic agility in the technology sector.

Aspect	Alignment with STS Theory	Challenges
**System Design Philosophy**	AI-integrated e-HRM tools (e.g. SAP SuccessFactors) support joint optimisation by enhancing both efficiency and employee engagement.	AI tools may prioritise technical accuracy over human usability, limiting accessibility and interpretability for HR professionals.
**Strategic Agility Support**	Scenario modelling facilitates proactive workforce planning aligned with dynamic business conditions, supporting strategic adaptability.	Tools may not fully capture non-quantifiable human factors (e.g. culture, emotions), leading to gaps in social context integration during decision-making.
**Human-Centred Innovation**	Real-time decision platforms reflect STS ideals by promoting transparent and inclusive decision-making across leadership and HR teams.	Over-reliance on AI outputs could marginalise human judgement and reduce participatory decision-making practices.
**Human-Centred Innovation**	Real-time decision platforms reflect STS ideals by promoting transparent and inclusive decision-making across leadership and HR teams.	Over-reliance on AI outputs could marginalise human judgement and reduce participatory decision-making practices.
**Learning and Development**	AI in e-HRM can personalise training pathways aligned with individual needs, respecting the social system’s diversity.	Lack of contextual sensitivity in automated learning systems may overlook social learning preferences and organisational culture.
**Organisational Collaboration**	Cross-functional interpretation of AI scenarios promotes shared understanding, aligning with STS emphasis on integrated social and technical collaboration.	Poor integration of cross-team feedback in AI models may hinder comprehensive scenario evaluation and reduce collective ownership of decisions.

**Table 16.** Alignment between STS theory and Digital Employee Experience (DEX) platforms in the technology industry.

Aspect	STS Theory Perspective	DEX Platform Application in Tech Industry	Challenges
**System Co-Design**	Emphasises the joint optimisation of social and technical systems.	DEX platforms (e.g. Microsoft Viva) integrate wellbeing, communication, and productivity tools.	Misalignment between tech features and employee needs may lead to disengagement.
**Personalisation & Engagement**	Recognises individual needs, learning styles, and social connections.	AI delivers tailored learning paths, feedback, and content (e.g. Salesforce Einstein AI).	Risks of depersonalisation if AI fails to consider nuanced human preferences.
**Transparency & Trust**	Advocates for systems employees can understand, trust, and effectively use.	Real-time analytics offer performance insights, yet demand clear communication and data use policies.	Lack of transparency in AI-driven decisions may cause distrust and anxiety among staff.
**Agility & Decision-Making **	Supports adaptive systems responsive to organisational and market shifts.	Scenario modelling and predictive analytics aid rapid strategic adjustments.	Over-reliance on AI predictions may overlook social or cultural workplace dynamics.
**Employee Autonomy & Satisfaction**	Encourages autonomy, meaningful work, and supportive environments.	Self-service hubs and personalised interfaces increase efficiency and satisfaction.	Data overload or poor UX design may overwhelm users and reduce perceived autonomy.
**Implementation Barriers**	Requires balanced integration with awareness of social impact.	DEX systems align tech with human experience for better retention and agility.	Privacy concerns, resistance to change, and skill gaps in e-HRM system use hinder effective uptake.

### Predictive workforce analytics for strategic agility, real time decision making in technology sector

The existing literature affirms the transformative potential of predictive workforce analytics in enhancing strategic agility and decision-making within the technology sector ( Ononiwu et al., 2024; Roul et al., 2024) as shown in Table 2. Research by Rahaman and Bari (2024) demonstrates that AI-integrated e-HRM systems support proactive talent management by forecasting attrition risks, identifying high-potential employees, and aligning workforce capabilities with evolving business strategies. These findings are further reinforced by case studies of global firms such as IBM, Accenture, and Tata Consultancy Services, which highlight the role of predictive analytics in improving retention rates, enabling agile workforce planning, and facilitating timely reskilling efforts to meet shifting market demands ( Singh, 2024; Sharma & Rastogi, 2025; Verma et al., 2023). Additionally, research by Thakurta (2025) extends this discourse to mid-sized firms, providing evidence that these technologies are both scalable and adaptable beyond large multinational enterprises. Collectively, the literature emphasizes that predictive analytics is pivotal in transforming HR functions from operational support roles into strategic leadership positions, particularly in dynamic environments characterized by shortened skill cycles and accelerated innovation timelines.

Despite these promising developments, several critical gaps and contradictions remain unresolved. Much of the current research predominantly focuses on large, resource-rich organizations operating in developed markets, thereby limiting the generalizability of findings to SMEs and technology firms in developing economies, where digital infrastructure and data maturity often lag behind ( Singh, 2024; Sharma & Rastogi, 2025). Additionally, while technical capabilities of predictive systems receive significant attention, ethical concerns, such as algorithmic bias, data transparency, and employee trust, remain substantially underexplored in the existing body of work. Contradictory evidence also emerges regarding the sustainability of these technologies. While some studies present short-term organizational gains following implementation ( Lakshmi et al., 2024; Rahaman & Bari, 2024), there is a noticeable lack of longitudinal research investigating long-term impacts on workforce agility, innovation capacity, and employee engagement.

Further research is required to address these gaps, particularly in exploring cross-cultural differences in adoption patterns and assessing integration challenges across diverse HR subsystems. Developing robust ethical frameworks to guide the responsible application of AI-driven workforce analytics is equally critical. Such efforts would help ensure that predictive workforce analytics not only enhances organizational agility but also aligns with principles of fairness, transparency, and long-term sustainability in a globalized technology landscape.

### AI-driven talent acquisition for strategic agility, real time decision making in the technology sector

The current literature clearly establishes AI-driven talent acquisition as a catalyst for strategic agility and real-time decision-making in the technology sector ( Hamadneh et al., 2024). This is shown in Table 3. Empirical studies and corporate case analyses provide compelling evidence of AI’s effectiveness in enhancing recruitment efficiency, reducing hiring timelines, and improving candidate-job fit. Research by Johnson et al., (2021) and Pandey et al., (2023) highlights the transformative impact of AI in automating candidate screening, mitigating selection bias, and predicting future job performance. These developments have been exemplified by major corporations such as Google, Microsoft, and Infosys, which have successfully integrated AI into their hiring processes to secure competitive talent advantages ( Albassam, 2023; Shandy et al., 2025; Valavanidis, 2023). Collectively, this body of work underscores AI’s role in supporting strategic agility by ensuring that organizations can quickly access and align talent with evolving business needs in dynamic environments.

Despite these positive developments, notable gaps remain in the literature, particularly concerning the long-term implications of AI-driven hiring. While current research demonstrates improvements in recruitment speed and accuracy ( Johnson et al., 2021; Pandey et al., 2023), there is insufficient examination of post-hire outcomes, including employee performance, retention, and adaptability in fast-evolving technological settings. Furthermore, although case studies from leading global firms illustrate successful applications ( Albassam, 2023; Shandy et al., 2025), the scalability and accessibility of AI recruitment systems for SMEs remain significantly underexplored. This raises questions about equitable access to advanced recruitment technologies, especially for firms operating with constrained resources in developing economies, where digital infrastructure may be less robust.

While much of the literature emphasizes AI’s potential to reduce human bias and promote diversity, contradictions persist regarding its practical outcomes. Farinu (2025) and Emma (2024b) argue that although AI systems may reduce explicit bias, ethical challenges related to algorithmic opacity and dataset representativeness remain unresolved. Empirical evidence is still lacking to demonstrate that AI consistently generates unbiased hiring results across varying demographic and cultural contexts. Additionally, limited research explores the compatibility of AI-driven hiring tools with local labor laws and recruitment practices in emerging markets, posing challenges to global implementation.

Further investigation is necessary to assess these unresolved issues comprehensively. Critical areas for future research include evaluating the psychological impact of AI-based hiring on candidate perceptions of fairness, transparency, and trust in automated systems. Addressing these concerns will be essential to achieving sustainable, ethical integration of AI into recruitment practices that foster both organizational agility and positive employee relations.

### AI-based learning and development for strategic agility, real time decision making in the technology sector

The current literature on AI-based L&D in the technology sector highlights its significant potential to personalize employee training, foster workforce agility, and support strategic decision-making as reflected in Table 4 ( Carreno, 2024; Dixit & Jatav, 2024). Studies such as those by Samuel et al., (2025) and Nyathani (2023) affirm that AI-driven platforms enhance learning efficiency through adaptive content delivery, real-time feedback, and predictive recommendations tailored to individual skill gaps. These adaptive systems are particularly valuable in technology-driven industries, where continual upskilling is critical to maintaining competitiveness. Case studies from global technology leaders like Amazon, IBM, and Infosys further illustrate how AI-based L&D platforms can align individual development goals with evolving organizational strategies, helping firms remain responsive to market shifts and innovation demands ( Liu, 2022; Sierszen & Drabek, 2024; Aithal & Rao, 2024).

Despite this promising evidence, the research landscape remains dominated by analyses of large multinational corporations with ample technological, human, and financial resources to deploy sophisticated AI learning infrastructures ( Awashreh & Hassiba, 2025; Aithal & Rao, 2024). This concentration creates a significant research gap regarding the feasibility, scalability, and effectiveness of AI-based L&D systems within SMEs. Given that SMEs form a substantial portion of the global technology sector, especially in emerging markets, there is limited empirical evidence to demonstrate whether similar benefits can be achieved in resource-constrained settings. Additionally, while qualitative insights highlight adaptive learning’s potential, there is a dearth of rigorous, longitudinal studies assessing its long-term impact on tangible business outcomes such as return on investment (ROI), innovation capacity, productivity growth, and employee retention.

A further critical issue emerging from the literature relates to the ethical challenges embedded in AI-driven L&D systems. Although personalization improves learning relevance ( Samuel et al., 2025; Nyathani, 2023), underlying algorithmic models may inadvertently perpetuate workplace inequalities by privileging content aligned with dominant behavioral profiles, thereby marginalizing alternative learning pathways. Research by Bahangulu and Owusu-Berko (2025) raises concerns about the lack of algorithmic transparency and insufficient governance over how employee learning data is collected, processed, and applied. These gaps expose learners to risks related to privacy, data misuse, and diminished autonomy. Moreover, while high engagement metrics from firms like IBM and Infosys suggest positive adoption ( Aithal & Rao, 2024; Pandita et al., 2025), this may not reflect the broader workforce, where digital literacy, learning cultures, and motivational factors vary significantly. There remains a contradiction in the assumption that technology-sector employees are uniformly motivated to engage with AI-powered learning, overlooking contextual barriers such as workload pressures, cultural resistance, or inadequate technological infrastructure.

These limitations underscore the need for expanded research on both the behavioral and structural enablers of successful AI-based L&D adoption. There is also scope to explore how AI learning platforms can promote interdisciplinary skill development, particularly vital in technology sectors that require integration between technical, creative, and strategic competencies. Addressing these gaps will be essential to ensure that AI-enhanced L&D supports not only organizational agility and innovation but also inclusive, ethical, and equitable workforce development across varied organizational settings.

### Strategic scenario modelling and strategic agility, real time decision making in the technology sector

The existing literature thoroughly explores the transformative role of AI-integrated scenario modeling in strategic human resource management (SHRM), particularly its ability to enhance decision-making in uncertain and rapidly evolving environments ( Nweke & Adelusi, 2025) as seen in Table 5. Studies by Pandey et al., (2023, 2024) confirm that AI-powered e-HRM platforms enable the simulation of complex workforce scenarios, allowing HR leaders to shift from reactive approaches to proactive, data-driven planning. These systems have become essential for technology-driven firms navigating volatile markets, as they provide strategic foresight in areas such as workforce composition, skills forecasting, and succession planning ( Patil, 2024; George, 2024; Kumari & Yelkar, 2022; Mahajan et al., 2024). Case examples from industry leaders like SAP and Cisco further demonstrate how predictive analytics embedded in scenario models facilitate alignment between HR strategies and broader organizational objectives, including digital transformation initiatives and hybrid work transitions (( Dutta et al., 2025; Pandey et al., 2023; Quimba et al., 2024).

Despite these advancements, the empirical scope of current research remains largely constrained to large, resource-rich corporations with advanced technological infrastructures ( Dutta et al., 2025; Pandey et al., 2023; Quimba et al., 2024). This focus presents a significant gap regarding the adoption, adaptation, and effectiveness of AI-driven scenario planning in small and medium-sized technology enterprises. Given the critical role of SMEs in global innovation ecosystems ( Osano, 2023), their exclusion from empirical investigations limits the generalizability of current findings. There is insufficient exploration of how organizational size, digital maturity, and resource constraints influence the implementation, operationalization, and strategic utility of AI-enhanced scenario planning. Moreover, while qualitative case studies highlight success in strategic agility, they often lack the methodological rigor of longitudinal, data-driven research required to substantiate claims of sustained organizational benefit.

Another prominent issue in the literature concerns the methodological and ethical dimensions of AI-driven scenario modeling. Although scenario modeling proved valuable during global disruptions such as the COVID-19 pandemic ( Rang & Sjöstrand, 2021), much of the cited evidence stems from anecdotal case studies and consultancy reports rather than rigorous peer-reviewed research. This overreliance on practitioner-driven narratives diminishes the robustness of the evidence base). Furthermore, there is limited discourse on the governance of predictive models, including critical aspects such as algorithmic transparency, bias mitigation, and accountability in decision-making. The risks of embedding flawed assumptions into simulations can lead to strategic misalignments, particularly in volatile regulatory or economic contexts where data lags compromise predictive accuracy ( Singhal et al., 2024). Contradictions persist regarding the practicality of real-time scenario planning in dynamic environments, especially for organizations without real-time data infrastructure or advanced AI literacy ( Youvan, 2024).

These limitations point to several areas for further research. There is a pressing need for empirical studies that evaluate how SMEs can leverage AI-driven scenario models despite resource constraints, as well as longitudinal research to validate the long-term strategic advantages of such systems. Additionally, future investigations should focus on the socio-technical dimensions of predictive modeling, including the development of governance frameworks that ensure fairness, model interpretability, and employee trust in AI-generated strategic recommendations. Addressing these gaps will help broaden the practical applicability of AI-integrated scenario modeling, ensuring that its benefits extend beyond large enterprises to a more diverse range of organizational contexts.

### Digital employee experience platforms for strategic agility and real-time decision-making in the tech industry

The reviewed literature on AI-driven digital employee experience platforms highlights their growing strategic significance in promoting employee engagement, productivity, and retention, particularly within the technology sector ( Pandey et al., 2023, 2024) as reflected in Table 6. Platforms such as Salesforce’s Einstein and Microsoft Viva exemplify how artificial intelligence can personalize employee interactions, optimize HR service delivery, and foster wellbeing through adaptive interfaces and predictive insights ( Bandella, 2025; Parmar, 2023; Ekandjo, 2024; Ekandjo et al., 2023). Supported by academic contributions, studies demonstrate that features like real-time sentiment analysis, automated feedback loops, and predictive analytics help organizations pre-empt employee disengagement, leading to lower turnover rates and higher satisfaction ( Ristola, 2024; Kafey, 2025). These case examples present compelling narratives about the value of AI in creating responsive and employee-centric workplaces. However, much of the existing research remains concentrated on large multinational firms with robust digital infrastructures and substantial financial resources ( Bashynska et al., 2023; Ekandjo, 2024). This creates a notable gap concerning the relevance, scalability, and practical challenges of implementing AI-driven DEX platforms in SMEs, particularly in resource-constrained environments.

Although the literature celebrates the potential benefits of AI-enhanced DEX platforms, significant contradictions and unresolved issues persist. A primary concern involves the ethical implications of leveraging AI for continuous employee monitoring and behavior prediction. While these platforms purport to enhance the employee experience ( Bandella, 2025; Parmar, 2023), they often blur boundaries between personal autonomy and organizational control, especially when predictive analytics are used to assess potential disengagement or attrition risks. Concerns about algorithmic opacity, data privacy, and the perceived surveillance aspect of AI systems are largely underexplored in empirical studies. These gaps underscore the need to examine not just technical efficacy but also employee trust, fairness perceptions, and consent mechanisms. Furthermore, while early evidence suggests positive outcomes in digital-forward firms, there is insufficient research on how varying organizational cultures, generational expectations, and technological readiness impact adoption and satisfaction levels with AI-driven DEX systems.

The reviewed studies also reveal a methodological limitation in the form of reliance on anecdotal or consultancy-driven reports rather than longitudinal, peer-reviewed empirical research. Without longitudinal data, it is difficult to validate claims about sustained improvements in employee engagement, wellbeing, or retention attributable to AI-driven interventions. Additionally, existing studies often generalize findings from specific high-performing corporate environments without considering the diversity of workforce needs in different organizational and cultural contexts ( Bandella, 2025; Parmar, 2023; Ekandjo, 2024; Ekandjo et al., 2023). Addressing these research gaps will require interdisciplinary studies that incorporate perspectives from psychology, ethics, technology adoption, and HRM. Future research should prioritize the development of robust governance frameworks to manage privacy risks, promote algorithmic transparency, and support equitable access to AI-enhanced employee experiences across various organizational types and workforce demographics.

### Comparison of literature findings with previous studies

The literature reviewed is compared with previous studies to identify similarities and account for any differences emerging in the findings.

### Comparative analysis of predictive workforce analytics in the technology sector

Contemporary findings on predictive workforce analytics closely align with earlier research emphasizing the strategic value of data-driven HR in enhancing organizational agility as shown in Table 7. For instance, Ononiwu et al., (2024), Roul et al., (2024) and Burnett and Lisk, (2021) observed that predictive modelling improves the alignment between HR functions and long-term business objectives, a view reaffirmed by recent studies that demonstrate predictive tools’ capacity to identify attrition risks, skill gaps, and training needs in advance. The application of AI by firms such as IBM and Accenture to forecast employee behavior and future skill requirements reflects earlier insights Singh (2024) and Lakshmi et al., (2024), who highlighted that analytics-integrated HR systems significantly enhance forecasting accuracy and decision-making precision. Both historical and current analyses underscore the central role of data in enabling agile talent strategies, particularly in dynamic sectors like technology, where rapid evolution of job roles and accelerating skill obsolescence demand anticipatory workforce planning ( Subrahmanyam, 2025; Gowrishankkar et al., 2025). This continuity in findings suggests a consistent developmental trajectory of predictive HR analytics as a foundational element of strategic human resource management.

More recent literature broadens the scope of predictive analytics beyond large multinational corporations to include mid-sized and regional firms. Earlier research predominantly examined companies with extensive data infrastructures and sophisticated analytic capabilities ( Zong & Guan, 2024; Sharma & Rastogi, 2025). In contrast, studies Lee and Low (2019) illustrate how mid-tier technology firms, particularly in Asia, increasingly leverage AI-enhanced predictive tools for timely recruitment and resource allocation. This evolution signals greater accessibility of analytics technologies, facilitated by cloud computing and modular electronic HR management systems. Furthermore, whereas early studies largely emphasized cost reduction and operational efficiency ( Saleh et al., 2024), contemporary research highlights predictive analytics as a catalyst for resilience, employee engagement, and sustained innovation ( Adewusi et al., 2024; Nyoni, 2025). These emerging perspectives reflect a maturing understanding of workforce analytics as not only a reactive operational instrument but also a proactive enabler of strategic agility across varied organizational environments.

### Comparative analysis of AI-driven talent in the technology sector

Recent advancements in AI-driven talent acquisition reflect the broader evolution of HR technologies documented in earlier studies, while also marking a clear shift toward deeper strategic integration and enhanced operational agility ( Hamadneh et al., 2024). This is well shown in Table 8. Foundational research by Hukkeri and Pol (2025) and Bhushan (2024) highlighted AI’s role in reducing recruitment bias, improving candidate-job fit, and enhancing quality-of-hire metrics through machine learning and natural language processing. These insights have been confirmed and expanded by more recent investigations and corporate case examples, including Microsoft and Infosys, which showcase capabilities such as real-time candidate analytics, behavioral assessments, and dynamic talent pooling ( Valavanidis, 2023; Learning et al., 2023; Andreu, 2024). A consistent theme across these studies is AI’s capacity to optimize hiring efficiency and refine precision in matching candidate skills with job requirements, an imperative in the technology sector where skill specificity is crucial.

Earlier research primarily concentrated on automating screening processes and mitigating algorithmic bias, whereas contemporary implementations by firms like Google and Microsoft demonstrate a more comprehensive, strategic deployment of AI tools ( Albassam, 2023; Shandy et al., 2025; Valavanidis, 2023). These systems extend beyond operational automation to generate predictive insights that inform broader talent management decisions, thereby fostering organizational agility and sustaining competitive advantage ( Hamadneh et al., 2024). Moreover, newer studies emphasize candidate engagement, inclusive hiring practices, and the integration of talent acquisition with learning and development initiatives. Infosys, for example, employs AI to recommend skill-building opportunities for candidates who are partially qualified, reflecting a shift from isolated AI applications toward holistic, adaptive talent ecosystems ( Learning et al., 2023; Andreu, 2024). This evolution addresses the growing need for real-time decision-making and continuous workforce alignment amid rapid technological disruption.

### Comparative analysis of AI-based learning and development in the technology sector

Recent findings on AI-driven L&D platforms reveal a marked shift from traditional, static training methods toward more adaptive, personalized, and performance-oriented approaches ( Carreno, 2024) as discussed in Table 9. The deployment of AI to curate real-time, individualized learning pathways—exemplified by Amazon’s machine learning system and IBM’s Watson-enabled “Your Learning” platform—reflects earlier research that highlighted the limitations of conventional training in rapidly changing industries ( Liu, 2022; Sierszen & Drabek, 2024; Rane et al., 2024b; Pandita et al., 2025; Awashreh & Hassiba, 2025). Studies by Dixit and Jatav (2024), Samuel et al., (2025), Nyathani, (2023) and Preechasin (2024) support these corporate examples, identifying personalized, data-driven content delivery as a critical factor in enhancing workforce agility. A consistent theme across these works is the improvement in employee engagement and skill alignment, demonstrating a shared understanding that tailored learning fosters organizational responsiveness. More recent research further advances this perspective by emphasizing AI’s predictive capabilities, which not only personalize learning but also anticipate future skill requirements—an evolution beyond earlier focuses on optimizing current performance ( Bobitan et al., 2024; Lokesh et al., 2024, April).

While earlier literature concentrated on AI’s role in facilitating upskilling and improving learning efficiency, recent corporate implementations provide deeper insights into its strategic significance. Infosys’s LEX platform, for instance, integrates real-time business demands with individualized learning journeys, a dynamic underemphasized in previous studies ( Aithal & Rao, 2024). Furthermore, contemporary findings include measurable outcomes such as increased employee satisfaction, internal promotions, and enhanced client delivery, offering quantitative evidence of AI-enabled L&D effectiveness ( Nyathani, 2023; Samuel et al., 2025). Earlier research predominantly featured theoretical analyses or small-scale observations. The addition of gamification and social learning components within platforms like IBM’s Watson-enhanced system introduces a behavioral dimension to AI learning strategies, which previous frameworks had not fully addressed ( Awashreh & Hassiba, 2025; Pandita et al., 2025). Collectively, these developments indicate a maturation of AI applications in L&D, evolving from experimental initiatives to foundational elements of strategic workforce development in technology sectors.

### Comparative analysis of strategic scenario modelling in the technology sector

Contemporary research on AI-powered strategic scenario modeling marks a clear shift from traditional workforce forecasting toward dynamic, simulation-based decision-making frameworks ( Nweke & Adelusi, 2025) as discussed in Table 10. Building on earlier studies that emphasized the importance of data-driven HR strategies, recent findings highlight how AI enhances the granularity and responsiveness of scenario planning ( Hao et al., 2024; Spaniol & Rowland, 2023; Pandey et al., 2023, 2024). The application of platforms such as SAP SuccessFactors and Cisco’s workforce simulations illustrates how machine learning can model multiple future states, reinforcing prior insights about the role of predictive analytics in strategic human resource management ( Dutta et al., 2025; Quimba et al., 2024). Notably, current research provides concrete examples of how scenario modeling informs decisions related to hybrid work models, reskilling initiatives, and organizational transformation—areas that earlier studies acknowledged but lacked empirical validation for. This practical alignment signifies a significant evolution from theoretical discussions toward demonstrable evidence of AI’s utility in fostering strategic agility ( Kumari & Yelkar, 2022; Mahajan et al., 2024).

Earlier literature often treated HR scenario planning as an isolated or infrequent activity, while contemporary practices integrate scenario modeling into continuous e-HRM operations ( De Alwis et al., 2022). For instance, Cisco’s use of simulations to align HR strategies with digital transformation reflects a shift toward ongoing, AI-driven strategic alignment, moving away from the static planning methods described by ( Pandey et al., 2023). The inclusion of external data sources in predictive models, exemplified by SAP’s pandemic response, also diverges from prior internal-only approaches, thereby broadening the contextual relevance of workforce scenarios ( Dutta et al., 2025; Rang & Sjöstrand, 2021). Additionally, Deloitte’s 2020 report underscores a critical development: organizations employing AI-enabled scenario planning adapted more quickly during crises, a benefit less emphasized in earlier research ( Schwartz, 2021; Ponce & Lustig, 2023). These distinctions highlight the increasing integration of AI into strategic planning processes, reflecting both technological advancement and a growing recognition of human capital as a crucial lever for organizational resilience.

### Comparative analysis of digital employee experience platforms in the technology industry

Recent findings on DEX platforms highlight their critical role in advancing strategic agility and enabling real-time decision-making within the technology sector ( Pandey et al., 2023, 2024; Biswas et al., 2024). This is well explained in Table 11. Case studies from leading firms such as Salesforce, Microsoft, and IBM demonstrate that AI-powered e-HRM systems significantly improve personalization, responsiveness, and employee engagement—factors that earlier studies had anticipated but seldom evidenced at scale. While foundational research acknowledged the value of digital HR platforms in streamlining administrative tasks and boosting employee satisfaction ( Bandella, 2025; Parmar, 2023; Chakraborty & Majumder, 2024; Bashynska et al., 2023), contemporary implementations reveal a more strategic orientation. For instance, Microsoft’s Viva platform incorporates AI-driven insights into employee wellbeing and productivity, moving beyond mere operational efficiency toward strategic workforce development. This reflects a transition from traditional automation-focused approaches to a more comprehensive, human-centric model that leverages behavioral analytics to enhance retention and performance ( Ekandjo, 2024; Ekandjo et al., 2023).

Earlier research primarily emphasized the theoretical benefits of digital HR transformation ( Zhang, & Chen, 2024), but indicates a maturing ecosystem where AI not only supports but actively shapes workforce strategies current evidence from technology industry leaders. Modern e-HRM systems employ real-time sentiment analysis and predictive modeling that extend previous assumptions about employee engagement tools by providing early-warning mechanisms for attrition and disengagement ( Soomro, 2020). This aligns with John and Hajam (2024) observation that proactive, data-driven engagement strategies substantially reduce turnover. What distinguishes current practices is the extensive scale and integration of these tools across organizational systems, exemplified by IBM’s deployment of Watson for personalized employee development ( Chakraborty & Majumder, 2024; Bashynska et al., 2023). These findings confirm earlier scholarly projections while illustrating a notable evolution toward intelligent systems capable of adapting dynamically to both individual employee needs and broader organizational objectives.

### Theoretical implications

The study examines relationship between literature review and the underpinning theoretical framework thereby identifying the persistent challenges and eminent gaps.

### STS theory and predictive workforce analytics in the technology sector

Predictive workforce analytics and strategic agility are closely interconnected, especially in the technology sector, where rapid innovation and shifting skill requirements demand real-time responsiveness ( Ononiwu et al., 2024; Nwoke, 2025) as discussed in Table 12. The integration of AI-enabled e-HRM systems into HR processes allows organizations to anticipate workforce challenges and address them proactively, thereby supporting agility through timely decision-making ( Pandey et al., 2023). These systems enable firms to identify skill gaps, forecast attrition risks, and reallocate resources toward innovation-driven areas, ensuring talent remains aligned with evolving strategic objectives ( Burnett & Lisk, 2021). However, this alignment is effective only when technical capabilities and human factors operate in harmony. According to STS Theory, the successful implementation of predictive analytics depends on their capacity to complement organizational culture, employee expectations, and collaborative workflows ( Thomas, 2024). Predictive tools must not only be technically robust but also accessible and interpretable by HR professionals, fostering human agency in decision-making while enhancing operational agility ( Leal-Rodríguez et al., 2023).

Despite the clear advantages, several challenges can hinder the alignment of predictive workforce analytics with strategic decision-making. A primary obstacle is the disconnect between technological potential and the social dynamics within organizations ( Okon et al., 2024). For example, AI-driven insights may encounter resistance if employees view these tools as intrusive or if leaders lack trust in algorithmic recommendations ( Ivchyk, 2024). Issues related to data quality and ethical considerations, such as bias in predictive models and insufficient transparency in data usage, can further undermine system credibility ( Sasmal, 2024; Zong & Guan, 2024). Additionally, smaller organizations often face resource limitations that restrict investment in advanced analytics or the training needed to interpret AI outputs effectively ( Ramya et al., 2024). Without proper integration into organizational structures and decision-making cultures, predictive analytics may remain underutilized or misapplied. STS Theory emphasizes that sustainable strategic agility arises from the co-design of technology and social practices, underscoring that true agility requires not only technological sophistication but also empowerment and trust within the workforce.

### STS theory and AI-driven talent acquisition in the technology sector

The alignment between STS Theory and AI-driven talent acquisition centers on the theory’s core principle of jointly optimizing technological tools and human systems to enhance organizational performance ( Thomas, 2024; Zhang, et al., 2025; Ing, 2024; Guest, et al., 2022). This is clearly shown in Table 13. AI-enhanced recruitment platforms such as Google’s qDroid and Infosys Nia exemplify this integration by leveraging machine learning algorithms to streamline candidate matching while embedding fairness and transparency within hiring processes ( Albassam, 2023; Shandy et al., 2025; Learning et al., 2023; Andreu, 2024). These platforms embody STS principles by automating routine recruitment tasks and simultaneously augmenting the decision-making capabilities of HR professionals. This shift enables recruitment teams to focus less on manual screening and more on strategic activities like evaluating cultural fit and engaging candidates, thereby aligning AI’s technical capabilities with social goals such as inclusion, equity, and employee well-being. Consequently, AI tools augment rather than replace human judgment, facilitating real-time, evidence-based decisions and fostering organizational agility in talent acquisition ( Johnson et al., 2021; Pandey et al., 2023).

Despite the strategic synergy between AI tools and the STS framework, practical challenges persist. A major concern is the opacity of AI decision-making, which can undermine trust among HR professionals if algorithmic outputs lack interpretability or if biases are not transparently managed ( Joshi, 2023, December; Madhuri & Kumar, 2025). Although companies like Google and Microsoft incorporate fairness checks into their systems, ethical issues surrounding algorithmic bias, data privacy, and the potential dehumanization of recruitment processes remain significant ( Fritts & Cabrera, 2021; Scatiggio, 2020). From the STS perspective, these challenges underscore the risk of over-reliance on technological systems at the expense of the human element. To fully embody STS principles, AI-driven recruitment technology must be designed with continuous user feedback, ethical oversight, and cultural sensitivity. Moreover, sustaining alignment with evolving organizational norms and labor market dynamics demands ongoing socio-technical calibration, balancing algorithmic efficiency with human empathy and contextual understanding.

### STS theory and AI-based learning and development in the technology sector

AI-based L&D systems closely align with the fundamental principles of STS Theory by integrating technological capabilities with human-centered needs within organizational contexts as shown in Table 14 ( Carreno, 2024). These platforms personalize training pathways using real-time performance data, skills profiles, and career aspirations, ensuring that learning experiences remain relevant and engaging ( Dixit & Jatav, 2024). This approach reinforces employee agency, boosts motivation, and promotes continuous upskilling ( Samuel et al., 2025; Nyathani, 2023) outcomes that directly reflect STS’s focus on human satisfaction and development. Companies such as Amazon, IBM, and Infosys demonstrate how AI tools can simultaneously optimize technical efficiency and social cohesion by facilitating internal mobility, fostering employee engagement, and driving collaborative innovation ( Liu, 2022; Sierszen & Drabek, 2024; Aithal & Rao, 2024; Awashreh & Hassiba, 2025). Such dual optimization enhances strategic agility, enabling organizations to respond quickly to technological disruptions while maintaining workforce alignment with evolving business goals.

Despite these benefits, integrating AI-based L&D systems with STS Theory presents notable challenges. The complexity inherent in AI algorithms may create opacity in decision-making processes, potentially diminishing trust if employees and HR professionals cannot understand or validate the outputs ( Reitgruber, 2023; Dixit, & Jatav, 2024). Moreover, an over-reliance on algorithmic recommendations might reduce opportunities for informal learning and peer-to-peer interactions, thereby undermining the social aspects of development that STS considers essential. Customizing learning at scale also requires substantial investment in data infrastructure, governance, and continuous oversight to prevent bias, protect data privacy, and preserve contextual relevance ( April & Daya, 2025; Samuel et al., 2025). Without deliberate efforts to bridge the social and technical dimensions, through transparent system design, stakeholder involvement, and adaptive feedback mechanisms, organizations risk deploying systems that, while technically robust, are socially misaligned and fall short of delivering their intended strategic value.

### STS theory and strategic scenario modelling in the technology sector

Strategic scenario modelling and real-time decision-making supported by AI-integrated e-HRM systems align closely with the principles of STS Theory by fostering balanced optimization of both technological capabilities and human elements ( Nweke & Adelusi, 2025) as reflected in Table 15. In the technology sector, where agility and speed are paramount, AI-powered simulations allow firms to forecast the impact of strategic decisions, such as organizational restructuring, hybrid work adoption, or talent investment, before implementation ( Patil, 2024; George, 2024). This proactive approach reflects STS’s emphasis on designing systems that enhance not only operational efficiency but also employee engagement and transparency in decision-making ( Ing, 2024; Guest, et al., 2022). Platforms like SAP SuccessFactors and Cisco’s predictive workforce analytics illustrate how technological innovation can drive strategic agility when integrated within organizational processes that value human judgement, culture, and collaboration ( Dutta et al., 2025; Quimba et al., 2024).

Despite these synergies, significant challenges hinder the full realization of the STS ideal in technology-driven HR systems. A key obstacle is the design and deployment of AI tools that may prioritize technical accuracy over human interpretability and inclusiveness ( Magliocca et al., 2024; Saba et al., 2025). When workforce simulations or predictive analytics lack transparency or user-friendliness, HR professionals may distrust the results, leading to weak integration in strategic planning. Furthermore, excessive reliance on technology risks diminishing the social dynamics essential for effective decision-making, including team input, leadership consensus, and contextual insight. While AI can predict turnover or skill shortages ( Dawson, et al., 2020, December; Szajna, & Kostrzewski, 2022) it cannot fully capture emotional, cultural, or interpersonal factors that influence employee behavior. These limitations highlight the ongoing need to evaluate and refine e-HRM systems to ensure they support, rather than supplant, the social fabric of organizations, in accordance with STS Theory.

### STS theory and digital employee experience platforms in the tech industry

The relationship between STS Theory and DEX platforms in the technology industry centers on their shared focus on aligning technological innovation with human needs to optimize organizational performance ( Thomas, 2024; Zhang et al., 2025; Kravchuk et al., 2024). This is illustrated in Table 16. STS Theory argues that organizations operate most effectively when social systems, comprising people, culture, and interactions, are designed to complement technical systems such as tools, processes, and technologies ( Ciriello, et al., 2024; Yu et al., 2023). DEX platforms enhanced with AI embody this principle by delivering solutions that improve operational efficiency while simultaneously enhancing employee engagement, satisfaction, and well-being ( Hammad & Abu-Zaid, 2024; Banerjee, et al., 2024). Features like AI-powered chatbots, real-time feedback mechanisms, and predictive analytics are integrated into user-friendly interfaces that address employees’ needs for autonomy, relevance, and personalization—ensuring that technology empowers rather than alienates the workforce ( Chadha, 2024; Satpathy et al., 2024; Parmar, 2023).

Applying the STS framework enables technology firms to design and implement DEX platforms that extend beyond functional utility to support human interaction, emotional engagement, and cultural cohesion. Platforms such as Microsoft Viva and Salesforce’s Einstein AI exemplify this approach by providing personalized experiences that foster continuous learning, wellbeing, and effective communication ( Bandella, 2025; Parmar, 2023; Ekandjo, 2024; Ekandjo et al., 2023). This synergy strengthens strategic agility and employee retention by allowing organizations to respond swiftly to change while maintaining a supportive work environment, thus fulfilling STS’s goal of co-optimizing technical and social systems. Nevertheless, challenges remain in achieving this balance. A primary concern is ensuring that AI-powered systems remain transparent, explainable, and trusted by employees, especially when deployed in performance evaluations or predictive modelling. Additionally, excessive reliance on automation risks diminishing meaningful human interactions and overlooking important contextual nuances. Data privacy issues, skill shortages in HR analytics, and resistance to technological adoption further complicate the integration of DEX platforms with STS principles, potentially limiting their strategic impact in fast-evolving digital workplaces.

### Practical implications

AI and e-HRM systems fundamentally transform how firms approach agility and decision-making by automating routine HR tasks, enabling real-time data flows, and providing predictive insights through tools such as workforce analytics, scenario modelling, and AI-driven recruitment. These capabilities facilitate faster, more informed decisions, reducing response times to market changes, enhancing workforce planning accuracy, and allowing leadership to focus on strategic rather than administrative HR functions. Consequently, technology firms can better align human capital with shifting business priorities, strengthening organizational resilience and adaptability.

The integration of AI-based tools in talent acquisition and performance management further improves efficiency and talent alignment. Intelligent algorithms streamline recruitment processes, improve candidate-job fit assessments, and reduce cognitive bias during early screening stages. In performance management, AI-powered continuous feedback systems enable dynamic goal-setting and personalized development plans. However, successful implementation demands careful attention to fairness, transparency, and user acceptance. Without effective communication and employee engagement strategies, there is a risk of distrust or perceived loss of autonomy, which can undermine employee engagement.

From a socio-technical perspective, technological innovation alone is insufficient. STS Theory stresses the necessity of co-optimizing technology with social systems, such as organizational culture, trust networks, and participatory practices. This requires including employees in system design and policy development, delivering regular AI literacy training, and implementing transparent data governance protocols. These measures build confidence and promote responsible AI use in decision-making. Absent such integration, even the most advanced systems may be underutilized or resisted, weakening their strategic potential.

Challenges, especially in developing economies, highlight systemic barriers like limited digital infrastructure, skills shortages, data privacy concerns, and cultural resistance to change. Overcoming these challenges requires targeted capacity-building programs, investments in digital infrastructure, and the creation of clear national and organizational policies on ethical AI usage. Firms must also prioritize partnerships with educational institutions to cultivate future-ready HR talent capable of effectively leveraging e-HRM systems.

For HR and technology leaders, these findings imply a shift in leadership competencies. Leaders now need both technological fluency and social intelligence to manage hybrid systems where human insight complements machine logic. This dual capability enables leaders to guide AI adoption in ways that enhance employee experience, innovation, and long-term strategic outcomes. Therefore, leadership development programs should include modules on digital transformation, AI ethics, and participatory design to prepare leaders for this evolved role.

This review concludes that while AI and e-HRM offer powerful tools for strategic agility and decision-making, their effectiveness depends on thoughtful integration with human systems, particularly within the complex dynamics of technology firms in developing economies. Policies and practices that harmonize technical innovation with social engagement are essential to ensure digital transformation efforts remain inclusive, ethical, and strategically aligned.

### Concrete policy recommendations

Embedding advanced digital capabilities into policy frameworks holds transformative potential for enhancing strategic agility and real-time decision-making within the technology industry. As competitive pressures intensify and innovation cycles shorten, both governments and corporate leaders must align policy with technological tools that enable prediction, adaptation, and swift response to rapid shifts in talent, markets, and technology landscapes. Real-world success stories from global technology firms demonstrate how embedding these capabilities into business models can produce tangible benefits.

Policies supporting predictive workforce analytics are critical in anticipating talent shortages, skill redundancies, and evolving labor market trends before they affect organizational performance. IBM offers a compelling example through its Watson Analytics-powered HR system, which predicts employee turnover and recommends retention strategies ( Anuradha & Rani, 2024; Singh, 2024). Governments can replicate this success by developing national workforce intelligence systems, advocating for data interoperability standards, and incentivizing investments in predictive analytics while simultaneously enforcing robust data privacy and ethical safeguards.

AI-driven talent acquisition benefits greatly from policy facilitation, enhancing firms’ responsiveness to market dynamics. Microsoft’s Azure-based recruitment platform has demonstrated the value of AI in recruitment by automating candidate screening processes and predicting role compatibility to meet fluctuating operational demands, particularly during high-demand retail seasons ( Valavanidis, 2023). National platforms that leverage AI for talent matching in high-growth technology sectors can be enhanced through regulation mandating algorithmic transparency and bias mitigation. Such policies can fast-track the deployment of skilled professionals into strategic roles, equipping firms to maintain workforce responsiveness amid volatility.

Integrating AI-based learning and development systems into workforce policies helps continuously align skills with rapidly changing technological demands. Microsoft’s AI-driven learning platforms, including Viva Learning, recommend role-specific upskilling paths and forecast future training needs based on industry shifts ( Khamis, 2024). Public-private partnerships, tax incentives, and co-investment grants can further encourage firms to implement such adaptive learning solutions at scale. These measures cultivate a digitally literate and agile workforce prepared to meet ongoing disruption.

Strategic scenario modelling tools further bolster agility by enabling organizations to simulate diverse future states and assess the impact of alternative decisions. Shell has successfully utilized AI-enhanced scenario modelling to navigate oil market volatility and climate change risks, providing valuable foresight for corporate strategy ( McCarthy, 2025). Policies that promote the widespread use of such platforms empower leaders to test assumptions, prepare contingencies, and navigate uncertainty with greater confidence. Governments can democratize access to such tools by supporting shared data repositories, establishing regulatory foresight centers, and offering targeted training initiatives that embed scenario planning into routine strategic processes, thereby enhancing sector-wide systemic resilience.

Supporting the development and adoption of DEX platforms is vital for real-time decision-making and operational agility. Google exemplifies this through its internal feedback and analytics platforms that continuously gather workforce engagement data to support real-time decision-making ( Ononiwu et al., 2024; Gade, 2021). Policy frameworks should establish national standards to safeguard employee wellbeing, ensure data ethics, and guarantee accessibility. Facilitating real-time workforce intelligence enables organizations to align human capital strategies with rapidly evolving business objectives.

Effective real-time decision-making also depends on robust digital infrastructure, continuous high-frequency data flows, and agile governance models. Estonia’s national e-Government infrastructure showcases how investing in scalable cloud architectures and real-time digital platforms can revolutionize governance, offering valuable lessons for fostering adaptive, data-driven policymaking in the technology sector ( Almutairi, 2025; Crompvoets et al., 2024). Governments should encourage interoperability among digital systems, supported by clear guidelines for real-time data use. Cross-sector data integration, regulatory sandboxes, and responsive policy adjustment mechanisms ensure technology firms can make timely, evidence-based decisions grounded in accurate, current information.

When combined within an integrated policy framework, these recommendations not only enhance strategic agility within the technology industry but also cultivate a resilient, future-ready digital economy capable of absorbing shocks and capitalizing on emerging opportunities. By learning from pioneering examples in the private sector, governments and firms alike can navigate ongoing technological disruption with agility, inclusiveness, and foresight.

### Recommendations for future research

Future research should place strong emphasis on the ethical design and interpretability of AI algorithms within e-HRM platforms to address persistent concerns about fairness, transparency, and accountability. Despite leading technology firms such as Google and IBM implementing bias mitigation strategies, scholarly investigation is needed to assess how effectively these measures ensure equity across varied demographic groups within diverse cultural and organizational contexts. Research should focus on how different levels of algorithmic transparency affect HR professionals’ trust in AI outputs and their consequent willingness to integrate these tools into strategic decision-making. Longitudinal studies examining the long-term effects of AI-driven recruitment and talent management systems on employee retention, satisfaction, and organizational culture would provide essential insights for developing more human-centered AI solutions aligned with STS theory.

Another vital avenue for research involves exploring the adaptability of AI-enabled e-HRM systems amidst rapid technological evolution and shifting workforce expectations. Given the technology industry’s dynamic nature, studies should investigate how AI tools can be flexibly reconfigured to respond to changes in organizational strategy, market conditions, and talent requirements. The role of continuous employee feedback in refining AI algorithms to maintain relevance and inclusivity also merits detailed examination. Comparative analyses between large multinational technology firms like Google or Microsoft and SMEs could uncover variations in adoption approaches, scalability challenges, and socio-technical integration. These investigations would advance theoretical understanding while offering actionable guidance for designing agile, ethically grounded, and user-responsive e-HRM systems tailored to the technology sector’s unique demands.

### Limitation of the study

One major limitation of this study lies in its reliance on secondary literature and theoretical frameworks, which may not fully capture the dynamic, real-time complexities of AI integration in actual workplace environments. While STS Theory provides a robust lens for understanding the interaction between humans and technology, it may not adequately account for the wide range of contextual variations across different technology firms, particularly those operating under distinct regulatory, cultural, or economic conditions. The review focuses primarily on leading technology companies such as Google, Infosys, and IBM, which are often better resourced to integrate ethical AI systems and sophisticated e-HRM platforms. This emphasis may inadvertently overlook the challenges faced by smaller enterprises or emerging tech firms with limited access to advanced AI infrastructure or expertise.

Another limitation stems from the study’s examination of data limited to the 2015–2025 period, which presents notable constraints, especially in a field as fast-evolving as AI-driven talent acquisition and e-HRM in the technology industry. While this ten-year window captures significant advancements in machine learning, algorithmic fairness, and digital HR practices, it may exclude more recent breakthroughs or shifts in strategic priorities emerging beyond early 2025. Given the rapid pace of innovation in AI and the increasing adoption of generative AI tools in HR functions, new paradigms, regulatory changes, and evolving user expectations are already redefining best practices and organizational responses.

The time-bound scope also constrains the study’s ability to anticipate long-term socio-technical transformations, especially as post-2025 developments in explainable AI, data governance, and human-AI collaboration frameworks mature. By not incorporating the most recent data or projecting forthcoming trends, such as the integration of emotion AI or globally debated AI ethics regulations, the study risks offering insights that may become outdated or less applicable in the near future. Therefore, while the 2015–2025 timeframe provides a solid foundation for historical analysis, it limits the study’s relevance for forecasting future trajectories and informing adaptive strategic models within the technology sector.

## Conclusion

Enhancing strategic agility and real-time decision-making in the technology sector requires a systemic policy shift that integrates digital intelligence throughout the entire workforce lifecycle. Predictive workforce analytics emerges as a foundational pillar in this transformation. By enabling organizations to forecast future talent needs, skill shortages, and workforce mobility trends, predictive analytics ensures that strategic planning is anticipatory rather than reactive. Integrating these capabilities into national employment frameworks and organizational strategies allows both private and public actors to make evidence-based decisions that align workforce capabilities with emerging technological frontiers.

AI-driven talent acquisition further reinforces strategic agility by transforming how firms source, assess, and onboard human capital. Unlike traditional, static recruitment models, AI-infused systems facilitate dynamic, real-time matching of candidates to roles based on evolving organizational requirements. This precision and responsiveness enable firms to scale their talent base rapidly, reduce hiring lead times, and maintain operational momentum amid fast-changing market conditions. Policy interventions that promote transparent, ethical, and inclusive deployment of these technologies will be critical to building trust and efficacy in AI-enabled hiring processes.

In tandem, AI-based learning and development systems create an agile internal environment where employee skills evolve continuously alongside technological demands. Personalized, adaptive learning platforms reduce skill obsolescence while empowering workers to take ownership of their professional growth. Aligning workforce development with strategic business objectives ensures internal talent pools remain agile and capable of supporting innovation. Supportive policy measures, such as subsidies, co-investment initiatives, and accreditation for digital learning platforms, can further strengthen the technology sector’s responsiveness to change.

Strategic scenario modeling offers a critical decision-making framework that enables technology firms to anticipate and navigate uncertainty with greater confidence. AI-enhanced modeling tools simulate complex, multidimensional futures, providing actionable insights to support informed strategic pivots. Encouraging the use of these tools through policy incentives and regulatory foresight mechanisms ensures organizations embed foresight thinking into their core planning activities, enhancing resilience and positioning firms to capitalize on opportunities arising from disruptive shifts.

Digital employee experience platforms play a central role in maintaining real-time situational awareness of workforce dynamics. By integrating feedback loops, performance dashboards, and engagement tools, these platforms generate continuous streams of actionable data that inform managerial decisions. Firms can respond quickly to dips in morale, productivity bottlenecks, or shifts in employee sentiment, thus maintaining agility in internal operations. Policymakers can promote the adoption of these platforms through digital innovation grants and by establishing standards that protect employee rights and ensure data transparency.

Finally, real-time decision-making across the technology sector depends on the integration of these elements into a cohesive digital strategy. Policy frameworks must support seamless data interoperability, rapid experimentation via regulatory sandboxes, and the widespread deployment of intelligent systems. Governments and industry leaders should co-create infrastructure that facilitates rapid data sharing, real-time analytics, and adaptive governance. By fostering an ecosystem where decision-making is informed, timely, and flexible, these policies help ensure the technology sector remains competitive and resilient in an era marked by perpetual innovation and disruption.

Collectively, these six pillars provide a comprehensive roadmap for future-ready workforce and enterprise strategies. Their integration into national policy and organizational practice offers a blueprint for building a technologically agile, resilient, and inclusive digital economy.

## Data Availability

There is no underlying data associated with this review. Repository name: The nomenclature and analysis scripts have been publicly archived at Tom Ongesa, N. (2025). Enhancing Strategic Agility and Real-Time Decision-Making in the Technology Sector: Exploring the Role of AI and e-HRM Systems – A Systematic Literature Review. Zenodo. https://doi.org/10.5281/zenodo.17378010 and are available under an open license. This study contains the following extended data: **
Figure 1:** Graphical abstract **
Figure 2:** The PRISMA flow chart **
Figure 3:** Conceptual Framework Data are available under the terms of the Creative Commons Zero “No rights reserved” data waiver (CC0 1.0 Public domain dedication)
