# Supplementary material for: Enhancing Strategic Agility and Real Time Decision-Making in the Technology Sector: Exploring the Role of AI and e-HRM Systems
Source: F1000Res. 2025 Nov 10;14:1238. [Version 1] doi: 10.12688/f1000research.167708.1 (PMC12775665; doi:10.12688/f1000research.167708.1)
Supplement: Peer review history

# Reviewer report: Reviewer response for version 1

**DOI:** 10.5256/f1000research.184842.r443891

**Published:** 2026-01-19

**Authors:** Bhanumathi P

**Affiliations:** 1 MS Ramaiah Institute of Management, Bangalore, Karnataka, India

The work has been carried out rigorously and it is emphasizing on the current trendy topic .

 The latest technology like AI and its impact on the HR process and e-HRM systems and the way it ensures real time decisions.

 The use cases related to the topic is appropriate an suffice the study .

Are the rationale for, and objectives of, the Systematic Review clearly stated?

Yes

Is the statistical analysis and its interpretation appropriate?

Yes

If this is a Living Systematic Review, is the ‘living’ method appropriate and is the search schedule clearly defined and justified? (‘Living Systematic Review’ or a variation of this term should be included in the title.)

Yes

Are sufficient details of the methods and analysis provided to allow replication by others?

Yes

Are the conclusions drawn adequately supported by the results presented in the review?

Yes

Reviewer Expertise:

AI in HRM

I confirm that I have read this submission and believe that I have an appropriate level of expertise to confirm that it is of an acceptable scientific standard.

# Reviewer report: Reviewer response for version 1

**DOI:** 10.5256/f1000research.184842.r441540

**Published:** 2026-01-06

**Authors:** Nick Hadjinicolaou

**Affiliations:** 1 Adelaide Institute of Higher Education, Adelaide, Australia

Replicability would be enhanced by providing: (a) the exact search strings used in each database, (b) the full MMAT scoring rubric or thresholds applied, and (c) a brief example or template of the data extraction sheet.

 Maybe at least one example.

 Regarding statistical analysis, The study is framed explicitly as a qualitative, thematic systematic review, with the primary synthesis conducted through thematic coding rather than meta-analysis.

 Quantitative reporting is descriptive only (e.g., counts of records at each PRISMA stage and the final number of included studies), which is consistent with the stated aims and heterogeneous study designs.

 The main inferences (that AI/e‑HRM support agility but face technical, ethical, and organizational barriers) are drawn from thematic patterns across studies, not from inferential statistics, which is appropriate for this evidence base.

 The main conclusions are broadly consistent with, and supported by, the results reported in the review, although a few claims could benefit from closer tying to specific strands of evidence.

 This review is not presented or structured as a Living Systematic Review, and there is no living-update method.

 The paper sets out a strong developed/developing economies contrast, but the synthesis still leans more heavily on advanced‑economy cases.

 The conclusions are broadly supported but occasionally over‑generalize (e.g., claims that AI‑e‑HRM “future‑proofs” organizations); temper these with explicit reference to the heterogeneity and quality of the underlying studies.

  

- The limitations section should more clearly acknowledge:
  - English‑language and database restrictions.
  - The predominance of case studies and cross‑sectional designs, limiting causal inference.
  - Potential publication bias toward successful implementations.
- Practice and policy recommendations are strong; label them clearly as “implications” and, where possible, tie each recommendation back to the thematic evidence (e.g., which cluster of studies underpins a given recommendation)

- Check for consistency in author‑year formatting (e.g., handling of “2024a/2024b”, month qualifiers like “January”, and multi‑citation strings) and ensure every reference in the list is cited in the text and vice versa.
- There are a few long paragraphs that could be split for readability, especially in the Introduction and thematic results; shorter paragraphs will make key arguments more visible to reviewers.

Ensure all acronyms (e.g., DEX, L&D, STS) are defined at first use and used consistently thereafter.

Are the rationale for, and objectives of, the Systematic Review clearly stated?

Yes

Is the statistical analysis and its interpretation appropriate?

Not applicable

If this is a Living Systematic Review, is the ‘living’ method appropriate and is the search schedule clearly defined and justified? (‘Living Systematic Review’ or a variation of this term should be included in the title.)

Not applicable

Are sufficient details of the methods and analysis provided to allow replication by others?

Yes

Are the conclusions drawn adequately supported by the results presented in the review?

Yes

Reviewer Expertise:

Use of Artificial intelligence in the strategy profession, Strategic Project Management, Project Management, Project Portfolio Management

I confirm that I have read this submission and believe that I have an appropriate level of expertise to confirm that it is of an acceptable scientific standard.

# Reviewer report: Reviewer response for version 1

**DOI:** 10.5256/f1000research.184842.r443895

**Published:** 2026-01-05

**Authors:** Mo'men Hani Mahmoud

**Affiliations:** 1 Middle East University, Amman, Jordan

Overall Assessment: This manuscript presents a comprehensive and timely systematic literature review examining the role of Artificial Intelligence (AI) and electronic Human Resource Management (e-HRM) systems in enhancing strategic agility and real-time decision-making within the technology sector. The topic is highly relevant given the increasing reliance on AI-driven HR analytics, workforce automation, and digital decision-support systems in volatile and innovation-driven environments.

 The study demonstrates strong conceptual grounding, clear thematic organization, and wide coverage of recent literature (2015–2025). The use of Socio-Technical Systems (STS) theory as the underlying framework is appropriate and well-aligned with the study’s dual focus on technological capabilities and human systems.

 Overall, the paper makes a meaningful contribution, but it requires moderate revisions to strengthen methodological transparency, reduce redundancy, and sharpen its theoretical and practical contributions.

 Points Requiring Clarification: search strategy and quality scoring.

Are the rationale for, and objectives of, the Systematic Review clearly stated?

Yes

Is the statistical analysis and its interpretation appropriate?

Yes

If this is a Living Systematic Review, is the ‘living’ method appropriate and is the search schedule clearly defined and justified? (‘Living Systematic Review’ or a variation of this term should be included in the title.)

Not applicable

Are sufficient details of the methods and analysis provided to allow replication by others?

Partly

Are the conclusions drawn adequately supported by the results presented in the review?

Partly

Reviewer Expertise:

Human Resource Management, Organizational Behaviour, and Organizational Performance.

I confirm that I have read this submission and believe that I have an appropriate level of expertise to confirm that it is of an acceptable scientific standard.
